# A bipolar disorder-associated missense variant alters adenylyl cyclase 2 activity and promotes mania-like behavior

**DOI:** 10.1038/s41380-024-02663-w

**Published:** 2024-07-13

**Authors:** Paromita Sen, Oskar Ortiz, Elena Brivio, Danusa Menegaz, Laura Sotillos Elliott, Ying Du, Clemens Ries, Alon Chen, Wolfgang Wurst, Juan Pablo Lopez, Matthias Eder, Jan M. Deussing

**Affiliations:** 1 Molecular Neurogenetics, Max Planck Institute of Psychiatry, 80804 Munich, Germany; 2 Institute of Developmental Genetics, Helmholtz Zentrum München, 85764 Neuherberg, Germany; 3 Department Stress Neurobiology and Neurogenetics, Max Planck Institute of Psychiatry, 80804 Munich, Germany; 4 Department of Brain Sciences, Weizmann Institute of Science, Rehovot, 76100 Israel; 5 Department of Molecular Neuroscience, Weizmann Institute of Science, Rehovot, 76100 Israel; 6 Scientific Core Unit Electrophysiology, Max Planck Institute of Psychiatry, 80804 Munich, Germany; 7 Chair of Developmental Genetics, Munich School of Life Sciences Weihenstephan, Technical University of Munich, 85354 Freising, Germany; 8 Munich Cluster of Systems Neurology (SyNergy), Munich, Germany; 9 German Center for Neurodegenerative Diseases (DZNE) site Munich, 81377 Munich, Germany; 10 Department of Neuroscience, Karolinska Institutet, Stockholm, 17177 Sweden

**Keywords:** Bipolar disorder, Neuroscience

## Abstract

The single nucleotide polymorphism rs13166360, causing a substitution of valine (Val) 147 to leucine (Leu) in the adenylyl cyclase 2 (ADCY2), has previously been associated with bipolar disorder (BD). Here we show that the disease-associated ADCY2 missense mutation diminishes the enzyme´s capacity to generate the second messenger 3’,5’-cylic adenosine monophosphate (cAMP) by altering its subcellular localization. We established mice specifically carrying the Val to Leu substitution using CRISPR/Cas9-based gene editing. Mice homozygous for the Leu variant display symptoms of a mania-like state accompanied by cognitive impairments. Mutant animals show additional characteristic signs of rodent mania models, i.e., they are hypersensitive to amphetamine, the observed mania-like behaviors are responsive to lithium treatment and the Val to Leu substitution results in a shifted excitatory/inhibitory synaptic balance towards more excitation. Exposure to chronic social defeat stress switches homozygous Leu variant carriers from a mania- to a depressive-like state, a transition which is reminiscent of the alternations characterizing the symptomatology in BD patients. Single-cell RNA-seq (scRNA-seq) revealed widespread *Adcy2* mRNA expression in numerous hippocampal cell types. Differentially expressed genes particularly identified from glutamatergic CA1 neurons point towards ADCY2 variant-dependent alterations in multiple biological processes including cAMP-related signaling pathways. These results validate *ADCY2* as a BD risk gene, provide insights into underlying disease mechanisms, and potentially open novel avenues for therapeutic intervention strategies.

## Introduction

Bipolar disorder (BD) collectively terms a group of chronic psychiatric disorders characterized by recurrent manic and depressive episodes. With a lifetime prevalence of 1%, BD is a leading contributor to the global burden of disease [1]. This group of mental disorders is accompanied by severely impaired psychosocial functioning and premature mortality [2]. The disease´s pathophysiology and etiology remain largely elusive. However, based on twin and family studies, a heritability of 60–85% has been estimated, which is comparably high among psychiatric disorders, suggesting a significant genetic component that interacts with other factors, such as adverse environmental exposure, to shape the disease risk [3–5]. Genome-wide association studies (GWAS) have identified numerous loci associated with BD over the past years [6–13]. Among these loci, the adenylyl cyclase 2 gene (*ADCY2*) has been identified as a potential risk gene for BD [14], a finding which has been replicated by the latest, and to date largest, BD GWAS meta-analyses [15, 16].

ADCYs are enzymes converting adenosine triphosphate (ATP) into the central intracellular signaling mediator 3’,5’-cyclic adenosine monophosphate (cAMP) and pyrophosphate. Their activity is controlled by heteromeric G proteins. Thereby, these enzymes are capable of integrating signals from various G protein-coupled receptors (GPCRs) which are conveyed onto downstream signaling pathways via the second messenger cAMP [17]. The family of mammalian membrane-bound ADCYs comprises 9 isoforms, which are classified according to their signaling properties. ADCY2, together with ADCY4 and ADCY7, belongs to the group II ADCYs, which are activated by Gαs and Gβγ but insensitive to Ca^2+^/calmodulin (CaM). Similar to all other ADCYs, ADCY2 is expressed in the central nervous system but displays a distinct and broader expression pattern compared to other group II ADCYs [18].

In the past decades, psychiatric research has particularly focused on neurotransmitter- and neuromodulator-related GPCRs as potential risk factors and drug targets for therapeutic intervention [19, 20]. In contrast, ADCYs, which are essential for the generation of the second messenger cAMP, have largely been ignored [21]. This is somewhat surprising as accumulating evidence from human [14, 22–24] and mouse [25–30] studies suggests an involvement of different ADCYs in psychiatric disorders including autism, schizophrenia, depression and BD.

**Fig. 1:**
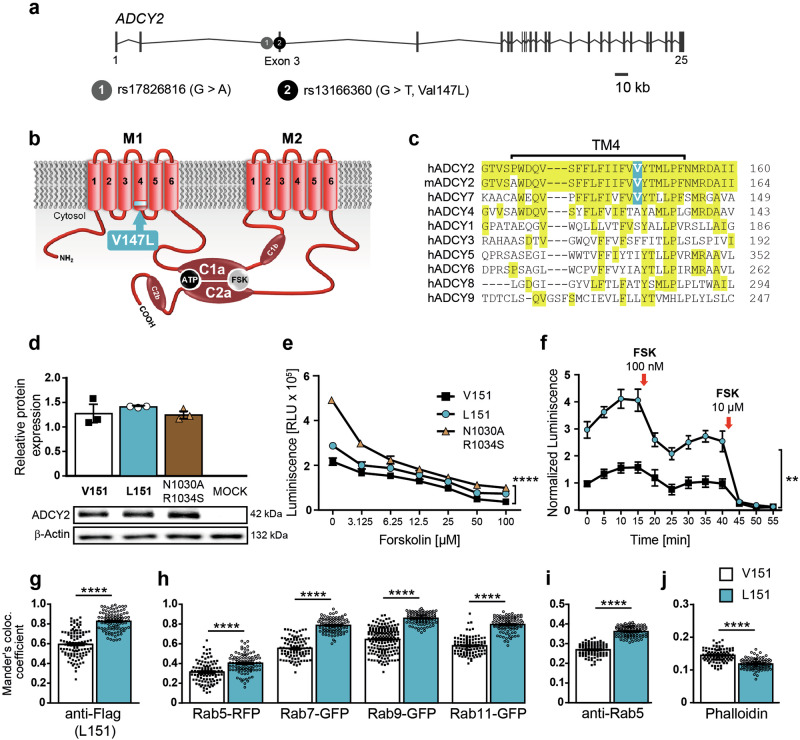
Consequences of Val147Leu substitution on ADCY2 activity and subcellular localization. **a** Organization of human *ADCY2* gene and localization of BD-associated SNPs rs17826816 and rs13166360. **b** rs13166360 causes a Val147 Leu substitution in the 4^th^ transmembrane helix (TM4) of the first transmembrane domain (M1). **c** Alignment of TM4 and adjacent regions of human and murine ADCY2 with human membrane bound ADCY paralogues. **d** Quantification and representative Western blot showing expression levels of ADCY2-V151, ADCY2-L151 and ADCY2-N1030A/R1034S in transiently transfected HEK293 cells (n = 3). **e** Forskolin (FSK) concentration-dependent stimulation of cAMP production by ADCY2 variants compared to the catalytically inactive variant ADCY2-N1030A/R1034S (n = 3; two-way ANOVA, ADCY2 variants: F_(1,14)_ = 127.6, ^****^p < 0.0001, concentration: F_(6,14)_ = 207.9, p < 0.0001, variants × concentration: F_(6,14)_ = 20.18, p < 0.0001). **f** Time course of cAMP production of ADCY2 variants at baseline and following repeated FSK stimulation (n = 3; two-way ANOVA repeated measures, ADCY2 variants: F_(2,3)_ = 98.37, ^**^p = 0.0018, time: F_(2,7)_ = 301^,^ p < 0.0001, variants × time: F_(12,18)_ = 22.17, p < 0.0001). Quantification of co-localization of HA-tagged ADCY2-V151 and HA-tagged ADCY2-L151 with: (**g**) FLAG-tagged ADCY2-L151, (**h**) RFP-tagged Rab5, GFP-tag**g**ed Rab7, Rab9, RAB11, (**i**) endogenous Rab5 and (**j**) phalloidin using Mander’s co-localization co-efficient. In all comparisons, unpaired t test, ^****^p < 0.0001. Each dot represents an individual cell. All data are presented as mean ± s.e.m..

In contrast to the vast majority of disease-associated SNPs, which are intergenic or intronic, one of the BD-associated SNPs - rs13166360 - is located in the 3^rd^ exon of the *ADCY2* gene (Fig. 1a). Hence, it encodes a missense mutation resulting in a Val to Leu substitution at position 147 (Val147Leu) within the 4^th^ transmembrane helix of the first transmembrane domain (Fig. 1b). Among ADCYs, this missense mutation is unique and its functional consequences are unclear. In ADCY2 homologs of different species, Val^147^ is fully conserved. Among other family members, however, it is only found in its closest relative ADCY7 (Fig. 1c). Based on ADCYs´ role as integrators of extracellular signals, it has been proposed that altered ADCY activity might have a more pronounced effect on BD susceptibility compared to functional variation on the level of individual neurotransmitter or neuromodulator receptors which provide a higher degree of redundancy facilitating compensatory processes [14].

Here we investigated the direct consequences of rs13166360 on ADCY2 function in vitro and in vivo. To validate *ADCY2* as a potential BD risk gene, we used heterologous expression to study protein function and generated a transgenic mouse model carrying the Val to Leu substitution. The latter approach allowed us to interrogate effects of the missense mutation with regards to BD-related endophenotypes and to probe its potential interaction with chronic stress as an environmental risk factor. Finally, we unraveled how the presence of the disease-associated SNP altered the transcriptional landscape in the ventral hippocampus, a brain structure involved in the regulation of emotionality and BD-related behavioral phenotypes.

## Materials and methods

### Cell culture experiments

Expression vectors pcDNA3.1-Adcy2-V151-HA and pcDNA3.1-Adcy2-V151-FLAG were cloned from mouse brain mRNA. pcDNA3.1-ADCY2-L151-HA, pcDNA3.1-ADCY2-L151-FLAG and the loss-of-function variant pcDNA3.1-ADCY2-N1030A/R1034S-HA were generated using the QuikChange II Mutagenesis Kit (Agilent). HEK293 and COS-7 cells were transfected with plasmid DNA using Lipofectamine 3000 according to the manufacturer´s protocol (ThermoFisher Scientific). Protein expression was assessed by Western blot and immunofluorescence. For details, refer to online Supplementary Information. Cell lines, originally purchased from the American Type Culture Collection, are regularly tested for mycoplasma contamination using PCR test.

### cAMP assays

cAMP levels of transfected cells were measured using the NanoBit® Protein:Protein Interaction System (Promega) and the cAMP-Glo^TM^ Assay (Promega) according to the manufacturer´s instructions. Fluorescence and luminescence signals were recorded using TriStar² LB 942 microplate reader. For details, refer to online Supplementary Information.

### Image acquisition and analysis

Images were taken using a Zeiss confocal microscope (LSM800) and digitalized using Zeiss Zen Microscope software and Fiji’s Image J. Image analyses were carried out using Fiji’s Image J and Image J plugin JACoP. Mander’s correlation coefficient was calculated and taken as a measure of the amount of co-localization.

### Animals

All animal experiments were conducted with the approval of and in accordance with the Guide of the Care and Use of Laboratory Animals of the Government of Upper Bavaria, Germany (Az.: 55.2-1-54-2532-142-2015). Mice were group-housed under standard lab conditions (22 ± 1 °C, 55 ± 5% humidity) and maintained under a 12 h light-dark cycle with food and water ad libitum. All experiments were conducted with adult male mice (age: 2–5 months).

### Generation of *Adcy2*^*V151L*^ mice

**Fig. 2:**
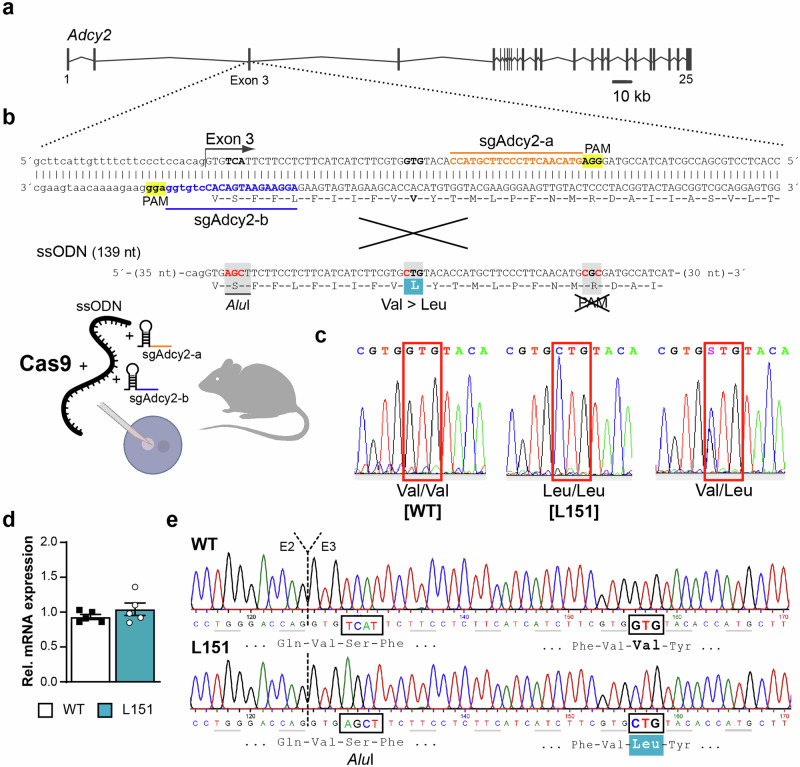
Generation of *Adcy2*^*V151L*^ mice. **a** Organization of the murine *Adcy2* gene. **b** CRISPR/Cas9-based strategy targeting exon 3 using sgRNAs sgAdcy2-a and sgAdcy2-b in combination with a ssODN to replace Val by Leu at position 151. **c** Representative electropherograms depicting possible genotypes of offspring from heterozygous breedings. **d** Quantification of *Adcy2* mRNA expression in the cortex of WT and L151 mice (n = 5 each) by RT-qPCR. **e** Representative electropherograms of *Adcy2* cDNA from WT and L151 mice covering the transition from exon 2 (E2) to exon 3 (E3). Highlighted are the *Alu*I restriction site and the Val151Leu substitution present in L151 mice.

Mice were generated using CRISPR/Cas9 technology as previously described [31]. Briefly, we designed two specific sgRNAs located 5´ (sgAdcy2-b) and 3´ (sgAdcy2-a) of Val151 in exon 3 of the *Adcy2* gene (Fig. 2a,b**)**. These sgRNAs were co-injected with a single-stranded oligonucleotide (ssODN) as a targeting vector into fertilized C57BL/6 J oocytes. The ssODN contained 2 silent mutations, one that added a restriction enzyme site (*Alu*I) for genotyping purposes and another one that obliterated the PAM sequence (NGG) so that the donor DNA was not cleaved by Cas9 (Fig. 2b). A heterozygous founder carrying the Leu151 mutation was used to establish the *Adcy2*^*V151L*^ line. Genotyping was done by PCR using primers Adcy2-I2-fwd 5´-CCA-CCG-CCA-ATG-CTT-CCT-GC-3´ and Adcy2-E3-rev 5´-AAC-AGG-TGC-TCC-TTG-GCC-CC-3´. For genotype determination, the 323 bp PCR product was either subjected to restriction endonuclease digest with *Alu*I or sequenced. Mice were backcrossed to C57BL/6 N for 5 generations and kept on a mixed C57BL/6 J × C57BL/6 N background. Potential off-target sites were identified using CRISPRscan and Cas-OFFinder allowing up to 2 mismatches [32, 33]. Potential off-target sites on chromosome 13 were amplified and sequenced using primers: Off-a2-1C: 5´-TAA-TGG-AGC-AGG-CCA-CTG-TC-3, Off-a2-1NC: 5´-TGT-CCC-CTC-AGA-ACT-TGA-TGC, Off-b6-1C: 5´-GCT-TGC-TGA-GAA-TGG-CTG-ATG, Off-b6-1NC: 5´-AGC-TGT-GCT-TGA-CTG-ACC-TC-3´, Off-b7-1C: 5´-TAC-CAC-AGG-ATG-GGG-GAA-GT-3´, Off-b7-1NC: 5´-TGA-GTC-CTC-AGA-GCC-CTA-CC-3´.

### Chronic social defeat stress (CSDS) paradigm

The chronic social defeat was performed as previously described [34]. For details, refer to online Supplementary Information.

### Behavioral testing

All behavioral tests were performed between 08:00 am and 12:00 pm in a room adjacent to the animal housing room. Independent batches of animals were used for basal behavioral characterization, cognitive assessment, amphetamine treatment, lithium chloride treatment, and chronic social defeat. The behavioral testing was conducted in the following order: open field test, dark-light box, forced swim test. Recording, tracking and scoring of animal behaviors was carried out using the automated video tracking system ANY-maze (ANY-maze 6.18; Stoelting Co, Wood Dale, IL, USA). All tests were performed by an experienced, blinded researcher and according to established protocols. For details, refer to online Supplementary Information.

### Amphetamine-induced hyperlocomotion

Mice were habituated in an OF arena by letting them freely explore the arena for 30 min. The next day, the mice were again introduced to the arena and allowed to freely explore it for 10 min. They were then injected intraperitoneally with amphetamine (4 mg/kg of body weight) or saline solution and placed back in the OF arena, which they were allowed to freely explore for the next 50 min.

### Lithium chloride administration

Lithium chloride (LiCl) was administered via the drinking water (600 mg/l) for 10 days. This dosage was chosen as previously described in the literature [35]. Behavioral testing started between day 10 and day 12, throughout which LiCl was continuously administered.

### Electrophysiology

The influence of the ADCY2 V151L substitution on hippocampal long-term potentiation was conducted as previously described [34]. Patch-clamp experiments were used for recording miniature excitatory postsynaptic currents (mEPSCs) and miniature inhibitory postsynaptic currents (mIPSCs) in the ventral hippocampus. For details, refer to online Supplementary Information.

### Determination of dopamine content by enzyme-linked immunosorbent assay

Brain regions of interest (PFC, striatum, nucleus accumbens, hippocampus) were isolated from freshly dissected brains. Brain tissue was homogenized in sterile PBS and dopamine content was determined using a dopamine ELISA kit (Aviva) according to the manufacturer´s protocol.

### Single cell RNA sequencing (ScRNA-seq)

Single-cell procedures were performed as previously described [36, 37]. For details, refer to online Supplementary Information.

### Gene Ontology Analysis

The gene ontology functional enrichment analysis was performed with g:Profiler (https://biit.cs.ut.ee/gprofiler/gost, version e106_eg53_p16_65fcd97) applying the Kyoto Encyclopedia of Gene and Genome (KEGG) biological pathways as a data source [38].

### Statistical analysis

Statistical analyses were performed using the commercially available software GraphPad Prism v5.0 (GraphPad Software, La Jolla, CA, USA). The sample size was chosen such that with a type 1 error of 0.05 and a type 2 error of 0.2, the effect size should be at least 1.2-fold of the pooled standard deviation. All results are presented as mean ± s.e.m.. All data was tested for normality with Shapiro-Wilk test. Behavioral phenotypic differences between two genotypes were evaluated with Student’s t test (two-tailed). Time-dependent measures were assessed with multi-factorial analysis of variance (ANOVA) with repeated measures. For experiments looking at the effect of pharmacological agents and for CSDS experiments, the effects of genotype and condition on all other behavioral and neuroendocrine parameters were assessed by two factorial ANOVA (two-way ANOVA). Whenever significant main or interaction effects were found by the ANOVAs, Bonferroni post hoc tests were carried out to locate simple effects. Statistical significance was defined as p < 0.05. All data were tested for outliers using Grubbs’ test. Animals were allocated to the experimental groups in a semi-randomized manner and data analysis was performed blinded to the group allocation.

## Results

### Val151Leu substitution in transmembrane domain 1 alters ADCY2 activity and subcellular localization

To test whether the SNP rs13166360 has a direct impact on protein function, we characterized ADCY2 variants with regards to expression, activity and subcellular localization. With the perspective of testing the Val to Leu missense mutation also in vivo, we focused on murine ADCY2, which shares 95% homology with the human protein but harbors an additional 4 amino acids at its N-terminus, shifting Val^147^ of the human protein to position 151 in the mouse ADCY2. We generated N-terminally HA- or FLAG-tagged wild-type (ADCY2-V151) and mutant (ADCY2-L151) variants of murine ADCY2, reflecting the human Val147Leu substitution. In addition, we generated an inactive ADCY2 variant (ADCY2-N1030A/R1034S) as a negative control. To study ADCY2 function by heterologous expression, we chose HEK293 cells as they endogenously express low levels of *ADCY2* mRNA (Supplementary Fig. 1). All three ADCY2 variants showed comparable levels of protein expression in transiently transfected HEK293 cells (Fig. 1d). However, comparison of ADCY2 activity by measuring cAMP production induced by the ADCY agonist forskolin (FSK) revealed reduced basal activity of ADCY2-L151 compared to the wild-type ADCY2-V151 variant as indicated by a significantly higher luminescence measured in the cAMP-Glo^TM^ assay, which is inversely proportional to cAMP levels. Nevertheless, the ADCY2-L151 activity was significantly higher than the background activity of endogenous ADCYs, as revealed by comparison to the inactive ADCY2-N1030A/R1034S variant (Fig. 1e). Similarly, assessment of cAMP production kinetics confirmed a reduced baseline activity of the ADCY2-L151 variant (Fig. 1f). Apart from the difference in baseline activity, the kinetics of cAMP production in response to different FSK concentrations or repeated stimulation thereby was comparable between both variants (Fig. 1e, f).

Since the difference in activity could not be attributed to different levels of protein expression or turnover, we hypothesized alterations in ADCY2 localization or trafficking. To this end, we directly compared the subcellular localization of ADCY2 variants and interrogated their localization in relation to different markers of the endosomal/lysosomal compartment. COS-7 cells were transiently transfected either with an HA-tagged variant of ADCY2-V151 or ADCY2-L151, each combined with a FLAG-tagged variant of ADCY2-L151 (Fig. 1g, Supplementary Fig. 2). Determination of Mander´s co-localization coefficient revealed a decreased degree of co-localization between ADCY2-V151 and ADCY2-L151 compared to the co-localization observed between the HA- and FLAG-tagged ADCY2-L151 variants. Moreover, ADCY2-L151 showed a significantly higher degree of co-localization with fluorescently tagged Rab5, Rab7, Rab9 and Rab11 compared to ADCY2-V151 (Fig. 1h, Supplementary Fig. 2). Similarly, antibody staining of transiently transfected cells for endogenous Rab5 revealed an increased co-localization with the ADCY2-L151 variant (Fig. 1i, Supplementary Fig. 2). In contrast, phalloidin staining of transiently transfected COS-7 cells revealed reduced co-localization of ADCY2-L151 with the plasma membrane compared to ADCY2-V151 (Fig. 1j, Supplementary Fig. 2). Taken together, these findings suggest a redistribution of the ADCY2-L151 variant from the plasma membrane to intracellular compartments.

### Homozygous mutant L151 mice show signs of a mania-like state

Next, we aimed at investigating the in vivo consequences of rs13166360 on mouse behavior. Therefore, we applied CRISPR/Cas9 technology to generate mice carrying the Val151Leu substitution. We used Cas-OFFinder and CRISPRscan [32, 33] to predict potential off-target sites (Supplementary Table 1). Selective amplification and sequencing of predicted off-target sites co-localized with *Adcy2* on chromosome 13, which might escape segregation during meiosis, did not reveal any undesired off-target effects. Breeding of heterozygous mice obtained wild-type *Adcy2*^*V151/V151*^ (WT) and homozygous mutant *Adcy*^*L151/L151*^ (L151) mice at mendelian ratio (Fig. 2a–c). Besides confirming the introduced Val151Leu mutation on the genomic DNA level, we substantiated equal expression levels of Ady2-WT and Adcy2-L151 mRNA in WT and L151 mice (Fig. 2d, e). Homozygous mutant L151 mice did not show any gross abnormalities.

**Fig. 3:**
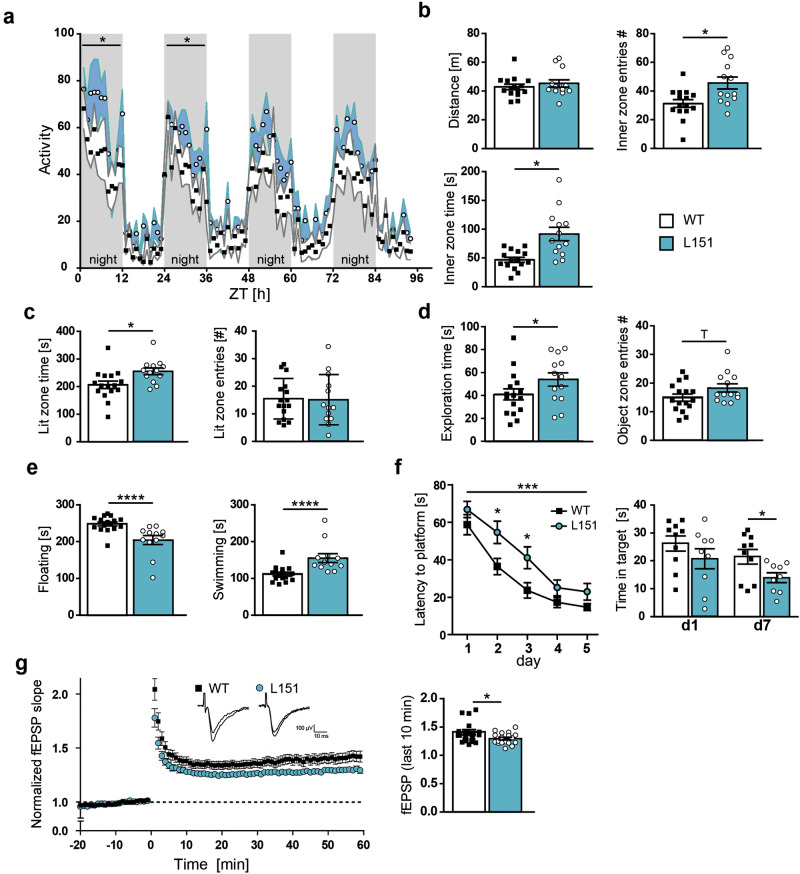
Behavioral assessment of L151 mice reveals signs of mania-like behavior and cognitive deficits. **a** Locomotor activity during adaptation to a novel home cage (WT n = 8, L151 n = 9; two-way ANOVA repeated measures, 1^st^ dark phase: genotype: F_(1,15)_ = 4.748, ^*^p = 0.0457, time: F_(2, 32)_ = 5.468, p = 0.006, genotype × time: F_(9,120)_ = 0.8536, p = 0.5687; 2nd dark phase: genotype: F_(1,14)_ = 5.215, ^*^p = 0.0385, time: F_(5,75)_ = 12.54, p < 0.0001, genotype × time: F_(11,154)_ = 0.9820, p = 0.4652). **b** Distance traveled, number of entries into (unpaired *t* test, ^*^p = 0.0199^)^ and time spent in the inner zone (unpaired t test, ^*^p = 0.035) of the open field test. **c** Time spent in and number of entries into the lit zone of the dark-light box (unpaired t test, ^*^p = 0.0344^)^. **d** Exploration of the object (unpaired t test, ^*^p = 0.0227) and object zone entries (unpaired t test, ^T^p = 0.087) in the novel object exploration test (**b–d**: WT n = 16, L151 n = 13). **e** Time spent floating and swimming in the forced swim test (WT n = 16, L151 = 12; unpaired t test, floating and swimming time: p < 0.0001 for both). **f** Latency to find the platform in the Morris water maze test (WT n = 10, L151 n = 9; two-way ANOVA repeated measures, genotype: ^***^p = 0.0003, F_(1,17)_^=^ 20.33, Bonferroni multiple comparisons test: day 2: ^*^p = 0.0478, day 3: ^*^p = 0.0187) and time spent in the target quadrant during memory retrieval (unpaired t test, ^*^p = 0.0325). **g** LTP at CA3-CA1 synapses in dorsal hippocampal slices from WT and L151 mice (n = 17 slices from 5 WT mice and 18 slices from 5 L151 mice; unpaired t test, ^*^p = 0.0298). Representative recording traces depict fEPSPs before and 60 min after LTP induction. All data are presented as mean ± s.e.m..

*Adcy2*^*V151L*^ mice were tested in a battery of behavioral paradigms to assess locomotion, exploratory and anxiety-related behavior, stress-coping strategies and cognitive performance. First, we assessed general activity and habituation to a novel environment over a period of 96 hours. In particular in the first two dark phases, L151 mice showed significantly higher activity compared to WT littermates (Fig. 3a). In the open field test (OFT), the distance traveled was not different between WT and L151 littermates. However, L151 mice entered the inner zone more often and spent more time in the center compared to WT mice (Fig. 3b). L151 mice also spent more time in the lit zone of the dark-light box (DaLi) (Fig. 3c). To further investigate object directed exploratory behavior, *Adcy2*^*V151L*^ mice were subjected to a novel object exploration (NOE) test. L151 mice showed increased time exploring the novel object and a trend towards more entries to the object zone (Fig. 3d). In the forced swim test (FST), L151 mice showed more active stress-coping behavior compared to WT littermates as indicated by decreased floating and increased swimming (Fig. 3e). To assess cognitive performance, *Adcy2*^*V151L*^ mice were subjected to the Morris water maze (MWM), showing that L151 mice were slower learners compared to their WT littermates (Fig. 3f) and displayed deficits in long-term memory as indicated by the reduced time spent in the target quadrant at day 7 (Fig. 3f). In view of the reduced performance in the MWM, we additionally evaluated hippocampal long-term potentiation (LTP) at CA3-CA1 synapses. Sixty minutes after high-frequency stimulation, L151 mice showed a significant decrease in LTP compared to their WT littermates (Fig. 3g). In summary, L151 mice revealed typical signs reminiscent of a mania-like state observable in rodent mania models as reflected by delayed ambulatory habituation to a novel environment, enhanced exploratory drive and cognitive deficits.

### L151 mice are more reactive to amphetamine, responsive to lithium treatment and show a shifted excitatory/inhibitory balance

**Fig. 4:**
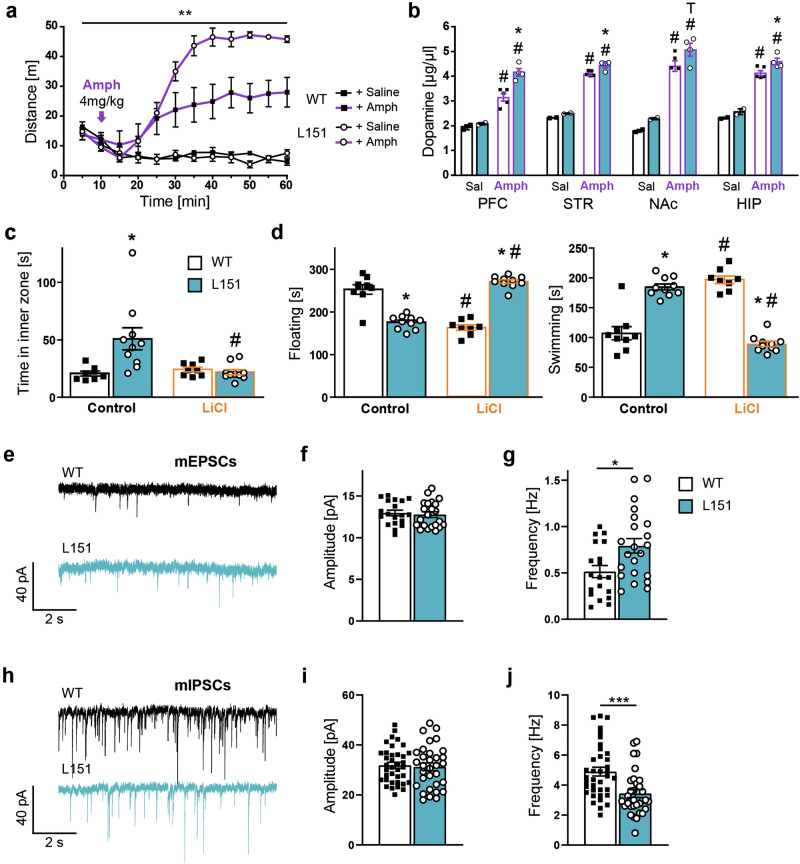
L151 mice show amphetamine hypersensitivity, lithium chloride responsiveness and a shift towards more synaptic excitation. **a** Locomotor activity following amphetamine treatment (WT n = 11, L151 n = 9; one-way ANOVA repeated measures, genotype: F_(3,7)_ = 8.1386, ^**^p = 0.0014). **b** Assessment of dopamine content in the prefrontal cortex (PFC), striatum (STR), nucleus accumbens (NAc) and hippocampus (HIP) following amphetamine treatment (WT n = 3; L151 n = 3; two-way ANOVA; PFC: genotype: F_(1,21)_ = 6.167, p = 0.0091, treatment: F _(1,21)_ = 21.63, p < 0.001, genotype × treatment: p = 23.514; STR: genotype: F_(1,21)_ = 8.914, p = 0.0141, treatment: F_(1,21)_ = 13.615, p = 0.010, genotype × treatment: F_(1,21)_ = 21.631, p = 0.017; NAc: genotype: F_(1,22)_ = 9.762, p = 0.610, treatment: F_(1,22)_ = 12.218, p = 0.012, genotype × treatment: F_(1,22)_ = 21.426, p = 0.047; HIP: genotype: F_(1,21)_ = 10.21, p = 0.0493, treatment: F_(1,21)_ = 6.514, p = 0.0121, genotype × treatment: F_(1,21)_ = 11.426, p = 0.0521). **c**, **d** Behavioral assessment of WT and L151 mice following 2 weeks of LiCl treatment (WT control n = 8, L151 control n = 10, WT LiCl n = 9, L151 LiCl n = 10). **c** Time spent in the inner zone of the open field test (two-way ANOVA, genotype: F_(1,31)_ = 8.641, p = 0.0062, treatment: F_(1,31)_ = 6.763, p = 0 .0141, genotype × treatment: F_(1,31)_ = 13.29, p = 0.001). **d** Time spent floating (two-way ANOVA, genotype: F_(1,33)_ = 4.530, p = 0.0409, treatment: F_(1,33)_ = 0.1745, p = 0.6788, genotype × treatment: F_(1,33)_ = 324.1, p < 0.0001) and swimming (two-way ANOVA, genotype: F_(1,33)_ = 169.4, p = 0.0409, treatment: F_(1,33)_ = 0.1745, p = 0.6788, genotype × treatment: F_(1,33)_ = 169.4, p < 0.000) in the forced swim test. **e** Representative traces, (**f**) amplitude and (**g**) frequency (unpaired t test, p = 0.0108) of patch-clamp recordings in the ventral hippocampus of miniature excitatory postsynaptic currents (mEPSCs; WT: n = 5 mice, 19 cells; L151: n = 4 mice, 22 cells). h Representative traces, (**i**) amplitude and (**j**) frequency (unpaired t test, p = 0.0005) of patch-clamp recordings in the ventral hippocampus of miniature inhibitory postsynaptic currents (mIPSCs; WT: n = 4 mice, 37 cells; L151: n = 4 mice, 32 cells). Bonferroni post hoc tests in (**b**–**d**): ^T^p = 0.0531, ^*^significantly different from WT of the same condition, p < 0.05; ^#^significantly different from the basal condition of the same genotype, p < 0.05. All data are presented as mean ± s.e.m..

Another characteristic mania-like behavior in animal models of BD is a hypersensitivity towards amphetamine [39]. As expected, amphetamine treatment (4 mg/kg) induced a strong increase in locomotor activity in *Adcy2*^*V151L*^ mice compared to saline treated animals. However, L151 mice showed an augmented response to amphetamine as indicated by enhanced locomotion compared to WT mice (Fig. 4a). Considering the difference in amphetamine sensitivity, we assessed dopamine (DA) levels in the prefrontal cortex (PFC), striatum (STR), nucleus accumbens (NAc) and hippocampus (HIP) of *Adcy2*^*V151L*^ mice 90 min after amphetamine application. Amphetamine treatment generally resulted in increased DA levels compared to saline treatment. Again, L151 mice displayed a stronger response to amphetamine as indicated by significantly increased dopamine levels in the PFC, striatum and hippocampus compared to WT mice (Fig. 4b).

Lithium is a commonly prescribed mood stabilizer for the treatment of mania, which is also able to reverse many of the behavioral abnormalities observed in genetic mouse models of mania [35]. Therefore, we assessed whether the mania-like state presented by homozygous mutant *Adcy2*^*V151L*^ mice could be reversed by lithium treatment. At first place, we confirmed previously observed signs of mania-like behavior in the untreated control group of L151 mice, i.e., increased inner zone time in the OFT (Fig. 4c) and decreased immobility as well as increased swimming in the FST (Fig. 4d). In the LiCl treated group, a normalization of behaviors of L151 mice was observed, which reached the level shown by untreated WT mice in the OFT (Fig. 4c) and in the FST (Fig. 4d).

A shift in the excitatory and inhibitory (E/I) synaptic balance has been suggested as a potential pathomechanism in BD, particularly playing a role in the manic phase [40]. Therefore, we recorded AMPA receptor-mediated miniature excitatory postsynaptic currents (mEPSCs) in CA1 pyramidal neurons of the ventral hippocampus (Fig. 4e). While the amplitude of mEPSCs was comparable between genotypes, the frequency was significantly increased in L151 compared to WT mice (Fig. 4f, g). We additionally recorded GABA_A_ receptor-mediated miniature inhibitory postsynaptic currents (mIPSCs) in WT and L151 mice (Fig. 4h). These measurements again revealed a comparable amplitude of recorded mIPSCs, but a significantly decreased frequency in L151 compared to WT mice (Fig. 4i, j).

The altered frequency of mEPSCs and mIPSCs suggest alterations in synaptic release probabilities. Therefore, we investigated the expression of the synaptic vesicle protein synapsin 1 (SYN1) as a presynaptic marker. Quantification of SYN1 levels by immunofluorescence in different subfields of the ventral hippocampus did not reveal any difference between WT and L151 mice with regards to SYN1 expression levels (Supplementary Fig. 3).

Together, with the enhanced reactivity to amphetamine and responsiveness to LiCl, the shift of the E/I balance towards increased excitation supports the notion that L151 mice present a mania-like state.

### Chronic social defeat stress induces a switch from a mania- to a depressive-like state in L151 mice

**Fig. 5:**
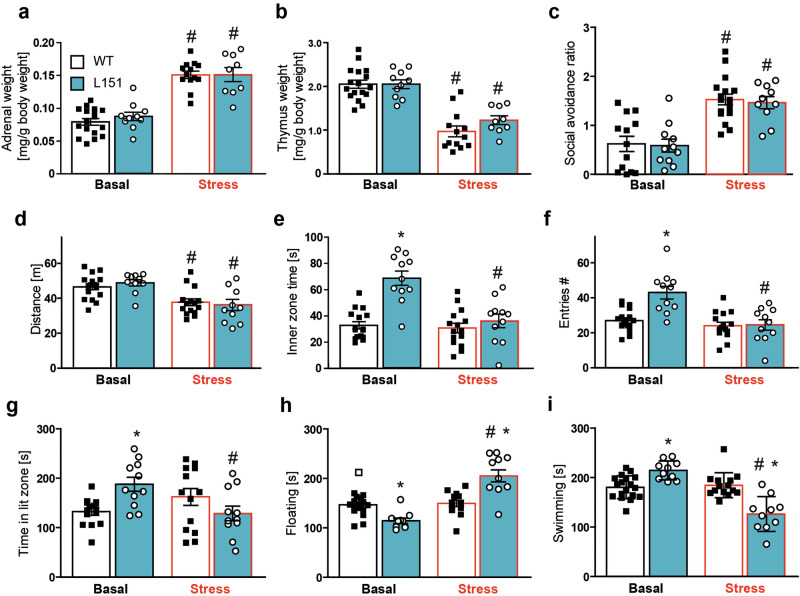
L151 mice show a switch in their behavioral state in response to chronic social defeat stress. **a–c** Validation of efficiency of chronic social defeat stress (CSDS) paradigm. Adrenal weight (**a**) (two-way ANOVA, stress: F_(1,45)_ = 99.06, p < 0.001), thymus weight (**b**) (two-way ANOVA, stress: F_(1,45)_ = 76.27, p < 0.001) and social avoidance ratio (**c**) (two-way ANOVA, stress: F_(1,47)_ = 43.88, p < 0.0001) of *Adcy*^*V151L*^ mice subjected to the CSDS. Distance traveled (**d**) (two-way ANOVA, stress: F_(1,49)_ = 24.33, p < 0.001), time spent in the inner zone (**e**) (two-way ANOVA, genotype: F_(1,48)_ = 32.97, p < 0.001, stress: F_(1,48)_ = 23.88, p < 0.001, genotype × stress: F_(1,48)_ = 19.02, p < 0.001) and entries to the inner zone (**f**) (two-way ANOVA, genotype: F_(1,50)_ = 11.56, p = 0.0013, stress: F_(1,50)_ = 19.72, p < 0.001, genotype × stress: F_(1,50)_ = 10.25, p = 0.0024) of the open field. **g** Time spent in the lit zone of the dark-light box (two-way ANOVA, genotype: F_(1,47)_ = 0.6375, p = 0.4286, stress: F_(1,47)_ = 1.142, p = 0.2906, genotype × stress: F_(1,47)_ = 10.73, p < 0.002). Time spent floating (**h**) (two-way ANOVA, genotype: F_(1,48)_ = 2.437, p = 0.1250, stress: F_(1,48)_ = 39.05, p < 0.001, genotype × stress: F_(1,48)_ = 34.55, p < 0.001) and swimming (**i**) in the forced swim test (two-way ANOVA, genotype: F_(1,48)_ = 2.972, p = 0.0912, stress: F_(1,48)_ = 33.15, p < 0.001, genotype × stress: F_(1,48)_ = 40.31, p < 0.0001). Groups: WT basal n = 16-17, L151 basal n = 10–14, WT stressed n = 10–15, L151 stressed n = 9–11. Bonferroni post hoc tests in (**a-i**): p < 0.05. ^*^significantly different from WT of the same condition, p < 0.05; ^#^significantly different from the basal condition of the same genotype. All data are presented as mean ± s.e.m..

Like most psychiatric disorders, BD involves genetic and environmental factors underlying its etiology. Particularly, the exposure to stressful life events is one factor that has been linked to the development of BD [41–43]. Therefore, we tested the behavioral consequences of chronic social defeat stress (CSDS) in *Adcy2*^*V151L*^ mice. Following 3 weeks of CSDS, *Adcy2*^*V151L*^ mice showed increased adrenal gland (Fig. 5a) and decreased thymus (Fig. 5b) weight independent of genotype, indicating the general efficiency of the CSDS. Similarly, exposure to CSDS entailed clear signs of social-avoidance behavior (Fig. 5c). In the OFT, stressed mice showed overall reduced locomotor activity independent of genotype (Fig. 5d). In the control group, L151 mice showed again signs of mania-like behavior as indicated by increased time spent in the center and an increased number of entries to the center of the OFT. However, while the performance of the stressed WT mice was indistinguishable from WT control mice, L151 mice displayed a significant reduction in the time spent in the inner zone (Fig. 5e) and in the number of entries to the inner zone of the OFT (Fig. 5f). Similarly, L151 mice responded to CSDS with reduction of the time spent in the lit zone of the DaLi box (Fig. 5g). Finally, we subjected *Adcy2*^*V151L*^ mice to the FST. Once more, we confirmed the previously observed enhanced active stress-coping behavior of L151 mice in the FST indicated by decreased floating and increased swimming time compared to WT littermates. Interestingly, L151 mice responded to CSDS by enhanced passive stress-coping behavior in comparison to unstressed L151 mice and WT littermates as reflected by increased floating (Fig. 5h) and decreased swimming (Fig. 5i) time. Taken together, the findings from the CSDS paradigm suggest a stress-induced switch from a mania- to a depressive-like state.

### ScRNA-seq uncovers Val151Leu-dependent transcriptional and cellular alterations in the ventral hippocampus

**Fig. 6:**
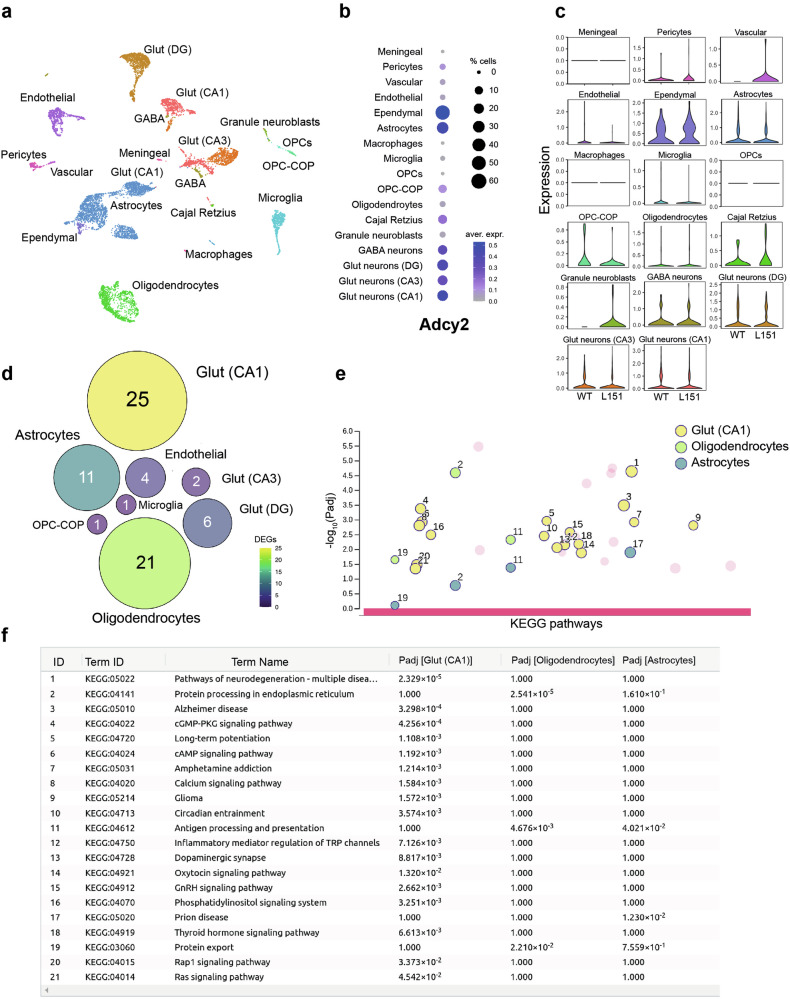
Single cell RNA sequencing of the ventral hippocampus of *Adcy2*^*V151L*^ mice. **a** Dimensionality reduction Uniform Manifold Approximation and Projection (UMAP) plot showing 33 clusters belonging to 17 main cell types. **b** Expression of ADCY2 in various cell types of the ventral hippocampus. **c** Violin plots comparing ADCY2 expression in different cell types of the ventral hippocampus of WT and L151 mice. **d** Number of differentially expressed genes between L151 and WT cells in various cell types. **e** Gene ontology functional enrichment analysis using Kyoto Encyclopedia of Gene and Genome (KEGG) biological pathways. **f** List of significantly enriched KEGG terms.

To resolve the cellular expression of *Adcy2* mRNA and to assess whether the Val151Leu missense mutation has any impact on downstream gene expression, we performed scRNA-seq on the ventral hippocampus (vHPC) of WT and L151 mice as previously described [36, 37]. We focused on the vHPC as a brain structure central for the regulation of emotionality which is potentially involved in BD-related behavioral phenotypes. Using the droplet-based 10x Genomics system, we collected 8477 individual cells that passed our quality control and identified 33 clusters belonging to 17 main cell types (Fig. 6a, Supplementary Fig. 4, Supplementary Fig. 5). Furthermore, we found that *Adcy2* mRNA is broadly expressed as it was detectable in various neuronal, glial and epithelial cells of the vHPC (Fig. 6b). To evaluate the impact of the ADCY2 missense mutation onto the transcriptional state of vHPC cell types, we performed differential gene expression analysis between WT and L151 conditions. In accordance with previous bulk protein expression experiments, *Adcy2* mRNA itself was not differentially expressed between cells derived from WT and L151 mice in any of the identified clusters (considering clusters with > 25 cells/cluster, Fig. 6c). In total, 71 differentially expressed genes (DEGs) were identified between WT and L151 mice and assigned to several clusters, i.e., CA1, CA3 and DG glutamatergic neurons, oligodendrocytes, OPC-COP, microglia, astrocytes, and endothelial cells (Fig. 6d, Supplementary Table 2).

To analyze the biological relevance of discovered genes, we chose the cell types with at least 10 DEGs (Fig. 6d) for a gene enrichment analysis using the Kyoto Encyclopedia of Genes and Genomes (KEGG) database (Fig. 6e). The largest number of enriched KEGG terms was identified from glutamatergic CA1 neuron-specific DEGs, while only 4 KEGG terms were found using astrocyte-specific DEGs, of which 3 were also identified using oligodendrocyte-specific DEGs (Fig. 6e). In sum, the gene enrichment analysis revealed a wide range of processes and pathways which are potentially contributing to the behavioral alterations observed in L151 mice (Fig. 6f).

Besides the analysis of gene expression, we used the scRNA-seq data as a proxy to evaluate the cellular composition of the ventral hippocampus. Therefore, we performed a binominal test for each of the identified clusters [44] in order to identify which of the observed numbers deviated from a binominal distribution indicating an enrichment of WT or mutant L151 cells. This analysis revealed significantly lower numbers of endothelial cells and DG glutamatergic neurons and OPC-COPs in the ventral hippocampus of L151 compared to WT mice. In contrast, the numbers of CA1 and CA3 glutamatergic neurons as well as OPCs were significantly higher in L151 mice (Supplementary Fig. 6a). Similar results were obtained by using a Fisher t test for each cluster to test enrichment/depletion of L151 cells versus WT cells (Supplementary Fig. 6b).

## Discussion

In recent years, ever larger GWAS have identified numerous BD-associated SNPs and respective gene loci. However, their role and contribution to disease etiology and pathophysiology remains largely unclear. Here we demonstrate that rs13166360 directly interferes with ADCY2 activity, diminishing its capacity to generate cAMP. Our results suggest that the impaired activity is not a consequence of reduced enzyme levels but is rather related to altered subcellular localization induced by the Val to Leu substitution in the 4^th^ transmembrane helix of transmembrane domain M1. Another example of a naturally occurring missense mutation among human ADCY family members is rs3730071, which leads to an alanine to serine substitution at position 674 of ADCY6. This mutation, which is located in the 7^th^ transmembrane helix of the M2 domain, also attenuates enzyme activity [45]. Interestingly, the highest frequency of polymorphisms and disease-associated mutations in human transmembrane proteins is found in transmembrane regions with non-polar to non-polar substitutions. Among these, Val to Leu substitutions are the most common ones [46]. Despite the conservative nature of this substitution, functional consequences, e.g., due to altered protein folding and protein-protein interactions, have been repeatedly reported for various proteins [47–51]. Thus, it is tempting to speculate that the disease-associated ADCY2 SNP might affect the proper interaction of the two transmembrane domains, which has been shown to be critical for correct targeting and functional assembly of ADCYs [52]. The fact that we demonstrate the reduced enzyme activity as a result of an altered subcellular localization of ADCY2-L151 only in a heterologous system involving overexpression of ADCY2 variants is admittedly a limitation of the study. However, current technical obstacles prevent a specific assessment in tissues or cells derived from *Adcy2*^*V151L*^ mice. On the one hand, there are no suitable ADCY2 antibodies available which would be a prerequisite to address endogenous ADCY2 localization in vivo, e.g., in neurons of ADCY2 mice. Simultaneous discrimination of both variants would even require to equip both variants with suitable tags for detection by immunocytochemistry and Western blot or for precipitation of interaction partners. On the other hand, the assessment of endogenous ADCY2-dependent cAMP levels or respective alterations caused by the V151L mutation is technically not practicable as the measurements will be masked by a high background caused by the other members of the ADCY family which cannot be completely blocked. The functional redundancy, overlapping and low expression, lack of isoform specific agonist and antagonist as well as the absence of reliable antibodies are intricacies inherent to the research on ADCY family members in vivo [17, 53–56].

Besides genetic associations of ADCYs [14, 22, 24, 26], general alterations in cAMP-dependent signaling pathways have been linked to psychiatric disorders [21, 57, 58]. In the case of BD, some studies have even suggested hyperactivation of cAMP-dependent pathways, which to some extent can be mitigated by lithium or carbamazepine treatment [59, 60]. Focused investigation of cAMP signaling-related genes revealed an association of PDE10A, DISC1 and GNAS with BD. Moreover, SNP × SNP interaction studies showed that variants in several of the analyzed cAMP signaling components including ADCY8 interact to increase the risk for BD [61]. Moreover, genetic mouse models targeting individual ADCYs have provided supportive evidence for their potential role in psychiatric disorders [25, 27–30]. However, none of those studies has addressed the causal involvement of disease-associated ADCY SNPs so far. To specifically study the in vivo consequences of rs13166360 on BD-associated endophenotypes, we established the *Adcy2*^*V151L*^ mouse line. Homozygous mutant L151 mice displayed signs of a mania-like state compared to WT littermates. These included hyperactivity, reduced anxiety-related behavior, increased exploratory drive and increased active stress-coping behavior. Hyperactivity of L151 mice was observed when mice were introduced into a novel home cage. However, this effect was only detectable during the first two nights but vanished with habituation to the novel setting. This is in accordance with observations in BD patients who also show increased locomotion in a novel environment as a result of delayed habituation [62]. In the light phase, no differences in activity were observed, which might also explain why L151 mice did not show hyperactivity in the OFT and DaLi. Nevertheless, L151 mice presented with significantly reduced anxiety-related behavior in these tests. Enhanced exploration of aversive areas in classical anxiety tests has been observed in many rodent models of mania reflecting enhanced risk-taking behavior and/or impulsivity, which is characteristic for BD individuals in a manic phase [35, 63, 64]. Similarly, the increased time spent on exploring a novel object in the NOE task mirrors signs of increased object exploration reported from patients with bipolar mania which discriminate BD from other psychiatric disorders like schizophrenia [62]. As many other psychiatric disorders, BD is also associated with significant cognitive impairments [65, 66] and mouse models of BD often show cognitive deficits [34]. L151 mice also displayed deficits in accomplishing spatial learning and memory tasks, which were additionally reflected by reduced hippocampal LTP indicating alterations in synaptic plasticity induced by the disease-associated ADCY2 variant. The involvement of Ca^2+^-dependent ADCYs in LTP has specifically been demonstrated earlier [67, 68]. However, increasing cAMP levels could also have a Ca^2+^ independent origin connected to direct activation of ADCY2 through the alpha subunit of various Gs protein-coupled receptors [69] [68].

The most common model of BD involves psychostimulant-induced hyperlocomotion. Amphetamine induces mania-like symptoms in healthy controls and aggravates symptoms in patients [70, 71]. Amphetamine-induced hyperactivity is considered as the ‘gold-standard’ rodent model of mania and is often reported in the context of genetic models of BD. Here we observed that amphetamine-induced hyperlocomotion is more pronounced in L151 mice compared to WT littermates. Concomitantly, we found higher DA levels in response to amphetamine in L151 mice in regions innervated by midbrain dopaminergic neurons. Currently, it remains unclear whether the detected differences are directly related to alterations in DA release kinetics or its turnover. Along these lines, hyperdopaminergic DA transporter knockdown mice also showed impaired habituation and increased exploratory behavior in novel environments [72]. DA mainly acts onto D1 and D2 receptor subtypes, which are positively and negatively coupled to ADCYs [73]. These results suggest a direct impact of altered ADCY2 activity on the dopaminergic system contributing to the observed behavioral phenotype. Lithium classically serves as a first-line medication for the treatment of BD patients during a manic episode. Accordingly, mouse models of BD particularly show reversibility of manic-like symptoms in response to lithium treatment [35, 39]. Chronic treatment of L151 mice was able to ameliorate behavioral alterations in the OFT and FST to the level of untreated WT mice, supporting previous reports connecting the effect of lithium at least partially to its direct or indirect impact on various ADCYs [74, 75].

We observed a specific shift in the frequency of mEPSCs and mIPSCs recorded in the ventral hippocampus indicating increased excitation in L151 mice and suggesting changes in the synaptic release probability. Accordingly, we did not observe genotype-dependent alterations in the presynaptic marker SYN1, suggesting no gross alterations in synaptic connectivity. Disturbances of E/I synaptic balance have been associated with various neurodevelopmental and neuropsychiatric disorders [76]. Although the role of such disturbances in BD is less explored, there are some genetic mouse models of mania which also show clear signs of E/I synaptic dysfunction [40]. SHANK3 overexpressing mice showed a reduction in mIPSC frequency and an increased amplitude of spontaneous EPSCs (sEPSCs) which was accompanied by a reduction of inhibitory and an increase in excitatory synaptic markers [77] [76]. To what extent the observed alterations in E/I synaptic balance in L151 mice are related to altered activity of GABAergic interneurons or even to an altered cellular composition of the ventral hippocampus is currently unclear but worth investigating in more detail in the future.

Our results indicate that *Adcy2*^*V151L*^ mice recapitulate many aspects of human mania. However, BD is a complex disease with patients alternating between manic and depressive episodes with euthymic or normal mood states between episodes. Relapse from one state to the other can be triggered by physical or sociopsychological stressors, although spontaneous alternations have also been reported. The development of mouse models spontaneously cycling between manic and depressive behavioral states has been challenging [78]. Chronic stress or even the treatment with glucocorticoids can trigger both manic and depressive episodes in BD patients [79, 80]. To test whether depression-like symptoms can be induced by an environmental trigger, *Adcy2*^*V151L*^ mice were exposed to CSDS. L151 and WT mice showed similar physiological signs indicative of a chronically stressed state. Their behavioral response, however, differed significantly. While anxiety-related and stress-coping behavior were largely unaffected in WT mice, L151 mice profoundly responded to the CSDS paradigm. The latter switched from a manic to a depressive-like state as indicated by increased anxiety-related behavior in the OFT and DaLi. Moreover, L151 mice transitioned from an active to a passive stress-coping strategy in the FST. Taken together, these results suggest that L151 mice are able to cycle between manic and depressive episodes following chronic stress exposure, closely resembling the switch between the manic and depressive state in BD patients.

Finally, scRNA-Seq revealed that *Adcy2* is not only widely expressed in the murine brain but is also present in a variety of different cell types, albeit the Val151Leu missense mutation did not have an impact on the expression of *Adcy2* mRNA itself in any of the identified cell clusters thereby confirming the bulk expression analysis in the cortex based on RT-qPCR (Fig. 2d). However, ADCYs are the central generators of the second messenger cAMP, which regulates numerous downstream pathways including transcription factors, such as CREB [81]. Thus, it is not surprising that the observed alteration in ADCY2 activity due to the Val151Leu substitution resulted in differential gene expression, particularly in CA1 glutamatergic neurons but also in oligodendrocytes and, to a lesser extent, in astrocytes. The gene ontology enrichment analysis of DEGs using the KEGG database yielded the highest number of affected processes and pathways in glutamatergic CA1 neurons. Among these, the term *cAMP signaling pathway* (KEGG: 040204) stands out as it is directly connected to ADCY2´s innate capacity to generate cAMP. Along these lines, *Rap1 signaling* (KEGG:04015) and *Ras signaling* (KEGG: 04014) are also linked to cAMP signaling via exchange proteins directly activated by cAMP (EPACs). EPACs, as intracellular sensors, respond to cAMP and act as specific guanine nucleotide exchange factors (GEFs) for small G proteins of the Ras family, such as Rap1 [82]. Similarly, *cGMP-PKG signaling* (KEGG04022) has been shown to play a role together with cAMP signaling in the context of *LTP* (KEGG: 04720) and long-term memory formation. Memory formation requires not only early-phase cGMP/PKG signaling but also late-phase cAMP/PKA-signaling [83]. Alterations in these pathways may indeed be causally linked to the reduced LTP and altered cognitive performance shown by L151 mice. cAMP and cGMP, in turn, can also modulate the activity of the 1,4,5-trisphosphate (InsP3) receptor, which is activated by InsP3 to trigger the release of Ca^2+^ from intracellular stores in the endoplasmatic reticulum, thereby linking the *calcium signaling pathway* (KEGG: 04020) to the *phosphatidylinositol signaling system* (KEGG:04070). Interestingly, the gene ontology enrichment analysis also identified terms like *dopaminergic synapse* (KEGG: 04728) and *amphetamine addiction* (KEGG: 04728), which is in line with our findings with respect to the increased amphetamine sensitivity and altered DA content in some of the analyzed brain structures of L151 mice, pointing towards disturbances related to the DA hypothesis of BD [84]. Nevertheless, the scRNA-Seq approach serves here as an initial screen which requires independent confirmation of DEGs and related pathways. In sum, the scRNA-seq analysis provides valuable entry points for a mechanistic understanding of alterations induced by the BD-associated ADCY2 variant which merit closer inspection in future studies.

In conclusion, the herein reported results provide evidence that the BD-associated Val147Leu missense mutation affects ADCY2 activity promoting mania-like symptoms in homozygous mice which, in combination with chronic stress exposure, are converted into a depressive-like state. These ADCY2-specific findings contribute to a growing number of human and mouse studies collectively pointing towards a prominent role of cAMP-related signaling pathways in psychiatric disorders including BD.

## Supplementary information

Supplementary Information

## Data Availability

All data needed to evaluate the conclusions in the paper are present in the paper and/or the Supplementary Materials. Additional data related to this paper may be requested from the authors. Raw sequencing data and annotated gene-barcode matrices are accessible on GEO using the accession number *GSE214733*.
