# Supplementary material for: A bipolar disorder-associated missense variant alters adenylyl cyclase 2 activity and promotes mania-like behavior
Source: Mol Psychiatry. 2024 Jul 13;30(1):97–110. doi: 10.1038/s41380-024-02663-w (PMC11649569; doi:10.1038/s41380-024-02663-w)
Supplement: Supplementary file 1 — Supplementary Information [file 41380_2024_2663_MOESM1_ESM.docx]

**Supplementary Information**

Supplementary Material and Methods

Supplementary Figures S1-S6

Supplementary Table S1 and S2

**Supplementary Material and Methods**

**Plasmid construction.** The cDNA encoding the C-terminal part of ADCY2 was amplified from mouse brain mRNA and cloned into the pcDNA3.1 expression vector via *BamH*I and *Xba*I. The 5´ sequence encoding the respective N-terminal part of ADCY2 was synthesized to possess an HA- or FLAG-tag at its N-terminus (Integrated DNA Technologies). It was inserted via *Hind*III and *BamH*I into the 5´ region of the expression vector containing the 3´ part of the cDNA to generate the final expression vectors pcDNA3.1-Adcy2-V151-HA and pcDNA3.1-Adcy2-V151-FLAG. The QuikChange II Mutagenesis Kit (Agilent) was used to introduce the V151L mutation resulting in pcDNA3.1-ADCY2-L151-HA and pcDNA3.1-ADCY2-L151-FLAG. The mutations N1030A and R1034S, which have been previously described to result in a loss-of-function, were also introduced into pcDNA3.1-ADCY2-V151-HA to generate pcDNA3.1-ADCY2-N1030A/R1034S-HA. All constructs were verified by sequencing (Eurofins Genomics).

**Cell culture and transfection.** HEK293 and COS-7 cells were maintained in Dulbecco's modified Eagle's medium (Invitrogen) supplemented with 10% FCS in 1% penicillin/streptomycin. For transfection, cells were cultured on poly-D-lysin-coated coverslips until they reached 80% confluency. Cells were transfected with plasmid DNA using Lipofectamine 3000 according to the manufacturer´s protocol (ThermoFisher Scientific).

**cAMP Assay.** The real-time measurement of cAMP was based on the PKA complex association and dissociation using the NanoBit® Protein:Protein Interaction System (Promega) according to the manufacturer´s instructions. HEK293 cells were transfected with the plasmid CAG-GFP and either pcDNA3.1-ADCY2-V151-HA, pcDNA3.1-ADCY2-L151-HA or pcDNA3.1-ADCY2-N1030A/R1034S-HA in combination with plasmids expressing the regulatory PKA subunit fused to LgBiT (LgBiT-PRKAR2A) and the catalytic subunit fused to SmBit (SmBiT-PRKACA). In addition, the cAMP-Glo^TM^ Assay (Promega) was used according to the manufacturer´s instructions to measure cAMP levels of cells transfected with either pcDNA3.1-ADCY2-V151-HA, pcDNA3.1-ADCY2-L151-HA or pcDNA3.1-ADCY2-N1030A/R1034S-HA following stimulation with ADCY agonist forskolin. Fluorescence and luminescence signals were recorded using TriStar² LB 942 microplate reader (Berthold Technologies Bioanalytic).

**RNA isolation and reverse transcriptase (RT) quantitative real-time PCR (qPCR)**. RNA was isolated from HEK293 or HT22 cells using the TRIzol reagent (Invitrogen) according to the manufacturer´s protocol. RNA from murine cortex was isolated using the Qiagen miRNeasy mini kit and QIAzol (Qiagen). cDNA was generated from total RNA using SuperScript™ II or SuperScript™ III reverse transcriptase (Invitrogen) and an oligo-dT primer according to the manufacturer’s instructions. The synthesized cDNA was utilized as template for qPCR, which was carried out in the Lightcycler 2.0 System (Roche Diagnostics) using the QuantiFast SYBR Kit (Qiagen). The following primers were used to quantify murine and human ADCY expression: Murine: Adcy1 fwd 5’-CCT TTT GGT CAC CTT CGT GT-3’, rev 5’-ACG CCA TAC ATG TTC ACA CC-3’; Adcy2 fwd 5’-CGA GTG TTC TCG CTG GTG AT-3’, rev 5’- TCC CTC ATG TTG AAG GGA AG-3’; Adcy3 fwd: 5’-AAA CAA GAT GGA GGC TGG TG-3’, rev 5’-TTG GAG GCA ATG ATG AGG T-3’; Adcy4 fwd 5’-GAG CCA ACA GTA CCC ACT GC-3’, rev 5’-CAG AGA GAA GCC ACC CAA AG-3’; Adcy5 fwd 5’-TCT CCA CTT GGC CAT CTC TC-3’, rev 5’-GAT ACA CTC CCG GGT CTC CT-3’; Adcy6 fwd 5’-CCC GTG TTC TTC GTC TAC A-3’, rev 5’-TTC AGC AGG GTA GTG TGT GC-3’; Adcy7 fwd 5’-ACA CTA CAT GCC CGA CAA CA-3’, rev 5’-GGT CAA ACT TCC CAA ACA GC-3’; Adcy8 fwd 5’-CCA GGC CTT CCT GGA GAC-3’, rev 5’-AGG TGC TCA TCC TCC ACA TT-3’; Adcy9 fwd 5’-CAT ATC TGA GGC CAC TGC AA-3’, rev 5’-ACC TCA AAG CCA GAA AGC AG-3’. Human: ADCY1 fwd 5’-TGA ACA TGC GTG TGG GTC TG-3’, rev 5’-GGC CAA GGT CAC ATC ATT GG-3’; ADCY2 fwd 5’-ATG TGG CAG GAG GCG ATG-3’, rev 5’-GGC TCA TGC AGT AGT AGG ACT CG-3’; ADCY3.1 fwd 5’-GCT TCA TGC GGC TGA CTT TC-3’, rev 5’-AAG ACC ACC AGC ACC AGC AG-3’; ADCY3.2 fwd 5’-TCC TCT ACC TGT GCG CCA TC-3’, rev 5’-TTC ATC TTC ACC TCC AGC GAC T-3’; ADCY4 fwd 5’-GAT GCG TCC ATC ACT GCT GA-3’, rev 5’-AGG GGT GGA GGT GGA CAC AG-3’; ADCY5 fwd 5’-CTG TCT GTC CTT CCC CGT CA-3’, rev 5’-TCA CGT TGT CAT GTT TCT GGA TG-3’; ADCY6 fwd 5’-GCT GCT GTT CCT CTG CAC CA-3’, rev 5’-CTG GAT GTA ACC GCG GGT CT-3’; ADCY7 fwd 5’-TGA CCA AGA TGC GCT CTA CGA-3’, rev 5’-GGC TGA AGG CAA TGA TGA TGA-3’; ADCY8 fwd 5’-AGT CTG CTG TCC TTG CCT GA-3’, rev 5’-AAG GGC AGT TCA GGG CTC CA-3’; ADCY9 fwd 5’-AAC TCA GAA CGG GCT CCT CA-3’, rev 5’-CCC TGC CCA CCT TCC CTT CT-3’. The following primers were used to amplify and sequence part of the murine *Adcy2* cDNA from exon 2 to exon 4: Adcy2-E2-fwd1: 5´-CTG GCC ATC TTC TTC GCC AT-3´ and Adcy2-E4-rev1 5´-CAG CTT GAT CCG GGA CTT GA-3´. Primers of housekeeping genes: Hprt-fwd 5´-TGG GCT TAC CTC ACT GCT TTC C-3´, Hprt-rev 5´-CCTGGT TCA TCA TCG CTA ATC ACG-3´; Gapdh-fwd 5´-GGG TCC CAG CTT AGG TTC AT-3´, Gapdh-rev 5´-CAT TCT CGG CCT TGA CTG TG-3´.

**Western blot.** For detection of differences in ADCY2 expression, HEK293 cells were transiently transfected with pcDNA3.1-ADCY2-V151-HA, pcDNA3.1-ADCY2-L151-HA or pcDNA3.1-ADCY2-N1030A/R1034S-HA. Subsequently, cells were lysed with RIPA buffer containing protease inhibitors (Roche Diagnostics). Protein samples were separated by 5% SDS-PAGE and transferred to 0.45-μm PVDF membranes (Millipore). The membranes were incubated with anti-HA-tag primary antibody (Abcam, Cat no 16B12, 1:1000) and a secondary HRP-conjugated antibody (Cell Signalling, Cat no 7074, 1:500). Chemiluminescence signals were visualized using a ChemiDoc station (BioRad) and analyzed using Image Lab (Bio-Rad).

**Immunohistochemistry.** Immunofluorescence staining was performed on transiently transfected COS-7 cells. Immortalized COS-7 cells were co-transfected with 200 ng of each of the respective plasmids: pcDNA3.1-ADCY2-V151-HA, pcDNA3.1-ADCY2-L151-HA, pcDNA3.1-ADCY2-L151-FLAG, GFP-Rab7 (Addgene plasmid #61803) ^1^, GFP-Rab9 (Addgene plasmid #12663) ^2^, GFP-Rab11 (Addgene plasmid #12674) ^2^, mRFP-Rab5 (Addgene plasmid #14437) ^3^. Cells were permeabilized with PBS-Triton X-100 0.1%, blocked at room temperature for 1 h in 5% BSA in PBS-Triton X-100 0.1%, and incubated overnight at 4 °C with the primary antibody. After washing with PBS-TritonX-100 0.01%, the selected secondary antibody, diluted in PBS-Triton 0.01%, was added, and incubated for 2 h at RT. After washing, cells were stained with DAPI and mounted with anti-fading fluorescence VectaShield medium (Vector Labs). Cells were stained with phalloidin (Thermo Fisher Alexa 488, Cat no A12379) and specific antibodies against HA-tag (Abcam, Cat no 16B12, 1:1000) and FLAG-tag (Sigma Aldrich, Cat no SAB4301135, 1:1000).

For immunohistochemistry on cryosections, animals were sacrificed using isoflurane and subsequently perfused with ice cold 1×PBS and 4% PFA. Dissected brains were post-fixed in 4% PFA overnight at 4°C, transferred to 30% sucrose in 1×PBS and incubated at 4 °C for 48 h. Brains were frozen on dry ice and cut coronally in 40 µm sections using a cryostat (Leica). Sections were collected in cryoprotection solution (25% Ethylene glycol, 25% glycerol, 50% ddH_2_O in 1×PBS) and stored at -20 °C until further use. For immunostaining, slices were washed 3× in 1×PBS, followed by blocking in 2% normal goat serum in 0.05% Triton-X100 and 1×PBS. Sections were incubated with primary anti-synapsin 1 antibody (Sigma Aldrich, Cat no AB1543, 1:500) at 4 °C under shaking overnight. After washing, sections were incubated with secondary antibody (goat anti rabbit Alexa 594, Invitrogen, Cat no A32740, 1:500) for 2 h at room temperature. Finally, sections were washed and mounted using Fluoromount-GTM mounting medium (Invitrogen, 15586276).

**Chronic social defeat stress (CSDS) paradigm.** Mice were subjected to chronic social defeat stress for 21 consecutive days. They were introduced into the home cage of a dominant CD1 for no longer than 5 min and were subsequently defeated. Once the test animals showed defeat, they spent 24 h in the same cage as the resident mouse, separated by a perforated partition that enabled sensory but not physical contact. Every day, experimental mice were exposed to a new unfamiliar resident. Control animals were single-housed in their home cages throughout the course of the experiment. All animals were weighed every 3 days and their fur status was evaluated. Control animals were single-housed in their home cages throughout the course of the experiment. All animals were handled daily, weight and fur status were assessed every 3-4 days. Behavioral testing was conducted during the last week of the CSDS paradigm in the following order: social avoidance test, open field test, dark-light box, forced swim test. At the end of the experiment, adrenal glands and thymus were collected and weighed.

**Behavioral tests.**

*Home cage activity.* Activity in a novel cage and subsequently in a familiar environment of the home cage was monitored by an automated infrared tracking system (Mouse-E-Motion 2.3.6, Infra-E-Motion). Animals were single-housed and the home cage activity was measured for consecutive 96 h.

*Open field test.* The open field test (OFT) was used to assess locomotor activity and anxiety-related behavior. The apparatus (50 × 50 × 50 cm) was made up of grey polyvinyl chloride (PVC) and evenly illuminated (10-15 lux). It was virtually divided into an outer and inner zone (15 × 15 cm). All mice were placed into a corner of the apparatus at the beginning of the trial and allowed to freely explore the apparatus for 30 min. Total distance travelled, time spent in the inner zone and number of inner zone entries were assessed.

*Dark/light box test.* The apparatus consisted of a protective dark (< 5 lux) chamber (15 × 20 × 25 cm) and an aversive brightly illuminated (700 lux) chamber (30 × 20 × 25 cm) that were connected by a small tunnel (5 × 7 cm). The mice were placed in the dark chamber and allowed to freely explore it for 10 min. Parameters assessed included time spent in the lit compartment and the number of lit compartment entries.

*Social avoidance test.* This test is typically carried out to assess if the test mice undergoing the CSDS paradigm have learned to avoid the aggressive resident CD1 mice. The test is carried out in the OF apparatus, which is evenly illuminated with 10 lux. The test mouse is first exposed to the apparatus with an empty wire cup on one side of the apparatus and allowed to freely explore the apparatus and the cup for 15 min. The amount of time the test mouse spends in the zone containing the wire cup is measured. A CD1 mouse is then placed in the wire cup and the test mouse is again allowed to freely explore the apparatus and the wire cup containing the CD1 mouse. The amount of time the test mouse spends in the zone containing the wire cup harboring the CD1 mouse is measured. The ratio of the time the test mouse spends exploring the empty wire cup to the time the test mouse spends exploring the wire cup containing the CD1 mouse is calculated and taken as the social avoidance ratio.

*Novel object exploration test*. We used the novel object exploration (NOE) test to assess object directed exploratory behavior. This test investigates exploratory behavior towards a novel object in a familiar environment, which is considered to reflect bipolar disorder associated traits ^4^. The open field apparatus (50 × 50 × 50 cm) was used for the testing paradigm and illumination was maintained at 10-15 lux. The test animals were first habituated to the OF apparatus for 10 min. Subsequently, during the object trial, a novel object made of Lego pieces was introduced on one side of the arena and the animals were allowed to explore the object for an additional 15 min. Entries into the object zone and the time spent with the object were assessed.

*Forced swim test.* The forced swim test (FST) was used to assess passive versus active stress-coping behavior in response to a stressful and unescapable environment. Each animal was placed into a 2 l glass beaker (diameter: 13 cm; height: 24 cm) filled up to a height of 15 cm with tap water (22 ± 1°C). The time spent floating, struggling and swimming was scored by an experienced observer, blinded to genotype or condition of the animals.

*Morris water maze.* The apparatus was a large cylindrical tank with a diameter of 150 cm and height of 40 cm filled with water up to 35 cm high. The tank was virtually divided into four quadrants: northeast, northwest, southeast and southwest. A circular platform (10 cm × 30 cm) was submerged just below the water at the center of the southwest quadrant. Large cues of different shapes were placed on the four walls of the testing room to help mice orient themselves while swimming and to find the platform. The test consisted of three stages: training, probe test and long-term probe test.

The training stage consisted of five training days with four trials per day. Each mouse was released from a different direction in each trial and trial sessions lasted for about 90 s with an inter-trial interval of 1 hr. If the mouse reached the platform before the session was over, it was allowed to sit on the platform for 10 s before drying and being returned to the home cage. If a mouse did not reach the platform within the 90 s, it was guided to the platform and allowed to sit there for 20 s. The latency to reach the platform was measured in every trial and average latencies per training day were calculated.

The probe test was conducted on day 6, 24 h after the last training session. The platform was removed, and the animals were released from the northeast quadrant. The animals were allowed to swim freely for 1 min and the time spent in each quadrant was measured. The long-term probe test was performed 7 days after the last training day and lasted for 1 min per mouse. The platform was removed from the southwest quadrant and the animals were released from the northeast quadrant. They were allowed to swim freely, and the time spent in each quadrant was measured.

**Electrophysiology.** Mice were anesthetized with isoflurane and decapitated. The brain was rapidly removed from the cranial cavity and, using a vibratome (HM650V, Thermo Scientific), 350 µm-thick coronal or horizontal slices containing the dorsal or ventral hippocampus, respectively, were cut in an ice-cold carbogen gas (95% O_2_/5% CO_2_)-saturated solution consisting of (in mM): 87 NaCl, 2.5 KCl, 25 NaHCO_3_, 1.25 NaH_2_PO_4_, 0.5 CaCl_2_, 7 MgCl_2_, 10 glucose, and 75 sucrose. Slices were incubated in carbogenated physiological saline for 30 min at 34°C and, afterwards, for at least 60 min at room temperature (23-25°C). The physiological saline contained (in mM): 125 NaCl, 2.5 KCl, 25 NaHCO_3_, 1.25 NaH_2_PO_4_, 2 CaCl_2_, 1 MgCl_2_, and 10 glucose. All measurements were conducted at room temperature. In the recording chamber, slices were superfused with carbogenated physiological saline (4-5 ml/min flow rate). Field excitatory postsynaptic potentials (fEPSPs) at CA3-CA1 synapses in the dorsal hippocampus were evoked by square-pulse electrical stimuli (50 µs pulse width) delivered via a bipolar tungsten electrode (50 μm pole diameter, ∼0.5 MΩ nominal impedance) to the Schaffer collateral-commissural pathway. fEPSPs were recorded using glass microelectrodes (filled with physiological saline, ~1 MΩ open-tip resistance) that were placed into the CA1 stratum radiatum. The intensity of voltage stimulation was adjusted in a manner to produce a fEPSP of ∼50% of the amplitude at which a population spike appeared. Recording data were low-pass filtered at 1 kHz and digitized at 5 kHz. Before and after LTP induction, a single stimulation pulse was delivered every 15 s to the neural tissue. LTP was induced by high-frequency stimulation (100 Hz for 1 s).

For patch-clamp experiments in the ventral hippocampus, infrared videomicroscopy was used to identify individual CA1 pyramidal neurons. Somatic whole-cell voltage-clamp recordings from these cells (-70 mV holding potential, >1 GΩ seal resistance, <20 MΩ series resistance, 10 mV liquid junction potential correction, 3 kHz low-pass filter, 15 kHz sampling rate) were performed with an EPC 10 amplifier (HEKA). For recording of AMPA receptor-mediated miniature excitatory postsynaptic currents (mEPSCs), the physiological saline contained picrotoxin (100 µM) and TTX (1 µM). Patch pipettes (3-5 MΩ open-tip resistance) were filled with a solution consisting of (in mM): 125 CsCH_3_SO_3_, 8 NaCl, 10 HEPES, 0.5 EGTA, 4 Mg-ATP, 0.3 Na-GTP, and 20 Na_2_-Phosphocreatine. For recording of GABA_A_ receptor-mediated miniature inhibitory postsynaptic currents (mIPSCs), NBQX (5 µM) and TTX (1 µM) were added to the physiological saline and the pipette solution contained (in mM): 140 KCl, 5 NaCl, 10 HEPES, 0.1 EGTA, 2 Mg-ATP, 0.3 Na-GTP, and 20 Na_2_-Phosphocreatine. 10 min after break-in to the cell, synaptic currents were recorded for 5 min. Offline analysis was performed using the Mini Analysis Program (Synaptosoft).

**Single cell RNA sequencing (ScRNA-seq).**

*Preparation of single cell suspension.* 10-12 weeks old male WT and L151 mice (n = 2/genotype) were sacrificed with a lethal dose of isoflurane and transcardially perfused with cold 1× PBS. Brains were quickly dissected and transferred to cold carbonated (95% O2, 5% CO2) artificial cerebrospinal fluid (aCSF: 87 mM NaCl, 2.5 mM KCl, 1.25 mM NaH_2_PO_4_, 26 mM NaHCO_3_, 10 mM glucose, 75 mM sucrose, 2 mM Mg^2+^, 1 mM Ca^2+^). From each brain, one slice of 1000 µm containing the ventral hippocampus (approximately -2.70 mm Bregma to -3.64 mm Bregma) was obtained using a VT1200/S Leica vibratome and the vHPC was manually dissected under a stereomicroscope. Tissue was then incubated in papain supplemented with DNase I (Papain Dissociation System, Worthington BC – LK003163) for 35 min at 37°C and triturated with a fire-polished glass pipette. The cell suspension was then filtered with a 30 µm strainer (CellTrics® 30 μm, sterile, Sysmex N. 04-004-2326) and layered over a gradient of ovomucoid protease inhibitor with bovine serum albumin (Papain Dissociation System, Worthington BC – LK003163). All steps were executed in cold and oxygenated aCSF. Cells were resuspended to a final concentration of ~1,000,000 cells/ml and loaded on the 10x Genomics Chromium Controller, v3 chips.

*Library preparation and sequencing.* The library was prepared using the 10x Genomics NEXT-GEM Single Cell 3’ Reagent Kits according to the manufacturer’s protocol. Molar concentration and fragment length of libraries were quantified using Bioanalyzer (Agilent High Sensitivity DNA kit – N. 5067-4626) and samples were pooled in equal molarity for sequencing. The pooled libraries were sequenced on a NovaSeq 6000 sequencer with paired-end asynchronous sequencing, 100 cycles with a depth of ~350 million reads per sample.

*Pre-processing and quality control.* Count matrices were obtained with the 10x Genomics CellRanger software and annotated on the mm10 reference set. Quality control, clustering and differential gene expression analysis were performed with the package Seurat v. 3.1.3 in R (version 3.6.3) ^5^, following best practice workflow in single cell data analysis and developers’ guidelines. Briefly, the package scran v. 1.14.6 (functions doubletCluster and doubletCells) was used to identify and eliminate putative doublets and multiplets ^6^. In addition, blood cells and droplets with less than 320 genes or more than 5,000 or mitochondrial gene content of more than 25% were removed. Following strict quality criteria, we had to exclude 1 sample from each genotype. This resulted in a dataset of 8,477 single cells, a median number of UMIs (unique molecular identifier) of 4,439, a median number of genes of 2,029, and median mitochondrial content of 0.058 per cell.

*Clustering.* For clustering, we selected the top 4,000 variable genes (FindVariableFeatures) after log-normalization and scaling (function NormalizeData, factor 10,000). Principal component analysis (PCA) was built on the scaled dataset (function ScaleData) using the identified variable genes (function RunPCA). We then clustered the cells using the first 25 principal components and a resolution of 1.2 by a shared nearest neighbor clustering algorithm (functions FindNeighbors and FindClusters). Finally, cells were plotted in the “Uniform Manifold Approximation and Projection” (UMAP) bidimensional space and the identity of the cell clusters was assigned by overlapping known marker genes from literature. This identified 33 clusters belonging to 17 main cell types: glutamatergic CA1 neurons, glutamatergic CA3 neurons, glutamatergic DG neurons, GABAergic neurons, granule neuroblasts, Cajal Retzius neurons, oligodendrocytes, OPC (oligodendrocytes progenitor cells), OPC-COP (committed oligodendrocytes progenitor cells), microglia, macrophages, astrocytes, ependymal, endothelial, vascular, pericytes and meningeal. We further validated the identity of the clusters by exploring the top representative markers for each cluster (FindAllMarkers with default settings).

*Differential gene expression analysis.* We used the function FindMarkers of Seurat and the statistical test MAST ^7^ integrated in the function to identify genes that were different between conditions. Only clusters with at least 25 cells per condition and genes expressed in at least 10% of cells per cluster were tested. Number of UMI was used as latent variable. Average gene expression per cluster was calculated using AverageExpression function on log-normalized scaled data.

**References**

1. Rowland AA, Chitwood PJ, Phillips MJ, Voeltz GK. ER contact sites define the position and timing of endosome fission. *Cell* **159**, 1027-1041 (2014).

2. Choudhury A*, et al.* Rab proteins mediate Golgi transport of caveola-internalized glycosphingolipids and correct lipid trafficking in Niemann-Pick C cells. *J Clin Invest* **109**, 1541-1550 (2002).

3. Vonderheit A, Helenius A. Rab7 associates with early endosomes to mediate sorting and transport of Semliki forest virus to late endosomes. *PLoS Biol* **3**, e233 (2005).

4. Perry W, Minassian A, Henry B, Kincaid M, Young JW, Geyer MA. Quantifying over-activity in bipolar and schizophrenia patients in a human open field paradigm. *Psychiatry Res* **178**, 84-91 (2010).

5. Stuart T*, et al.* Comprehensive Integration of Single-Cell Data. *Cell* **177**, 1888-1902 e1821 (2019).

6. Lun AT, Bach K, Marioni JC. Pooling across cells to normalize single-cell RNA sequencing data with many zero counts. *Genome Biol* **17**, 75 (2016).

7. Finak G*, et al.* MAST: a flexible statistical framework for assessing transcriptional changes and characterizing heterogeneity in single-cell RNA sequencing data. *Genome Biol* **16**, 278 (2015).

**Supplementary Figures**


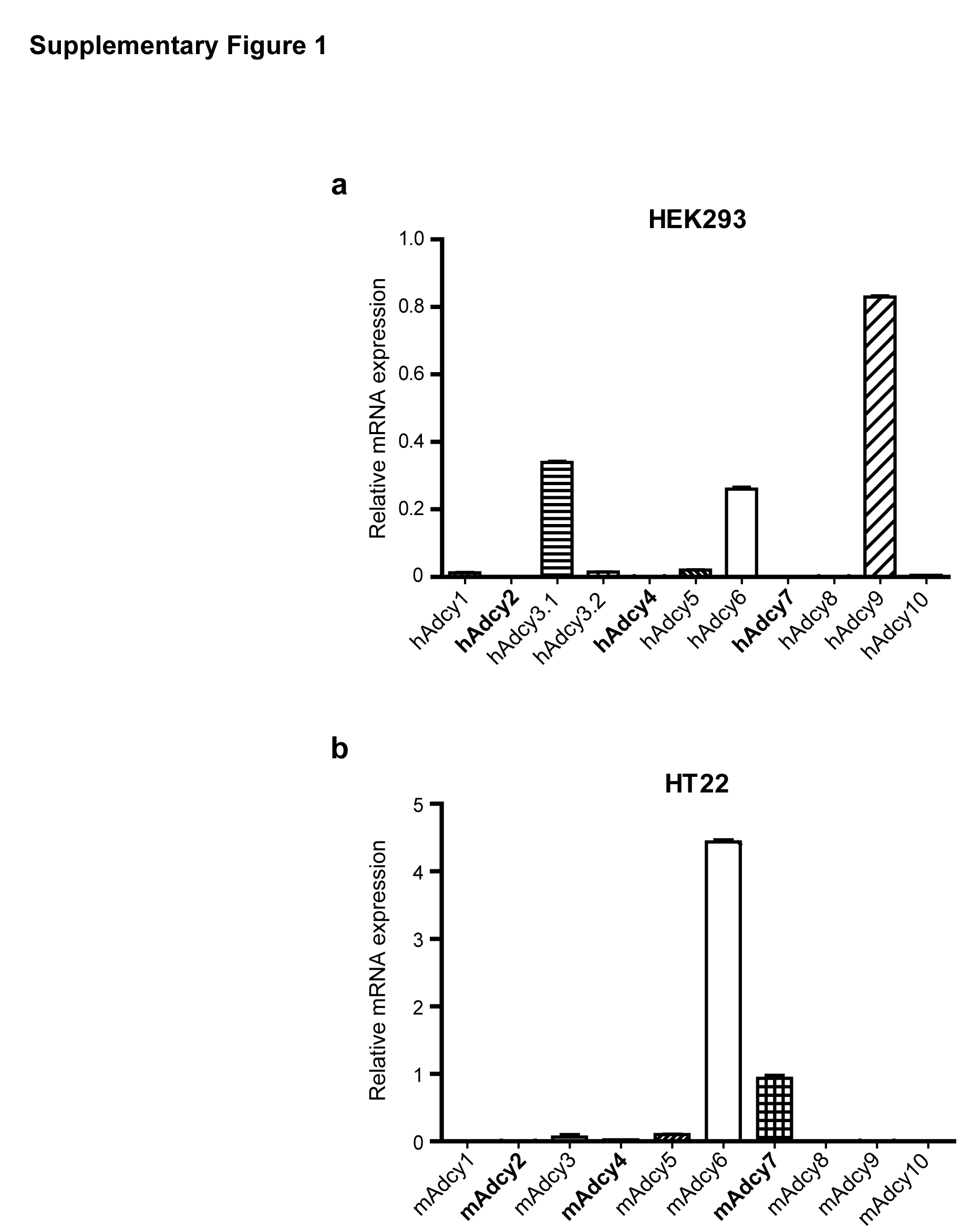


**Supplementary Fig. S1 Analysis of ADCY expression in HEK293 and HT22 cells.** Relative expression of human and murine ADCYs determined by RT-qPCR in HEK293 (a) and HT22 (b) cells. Group II ADCYS are depicted in bold.


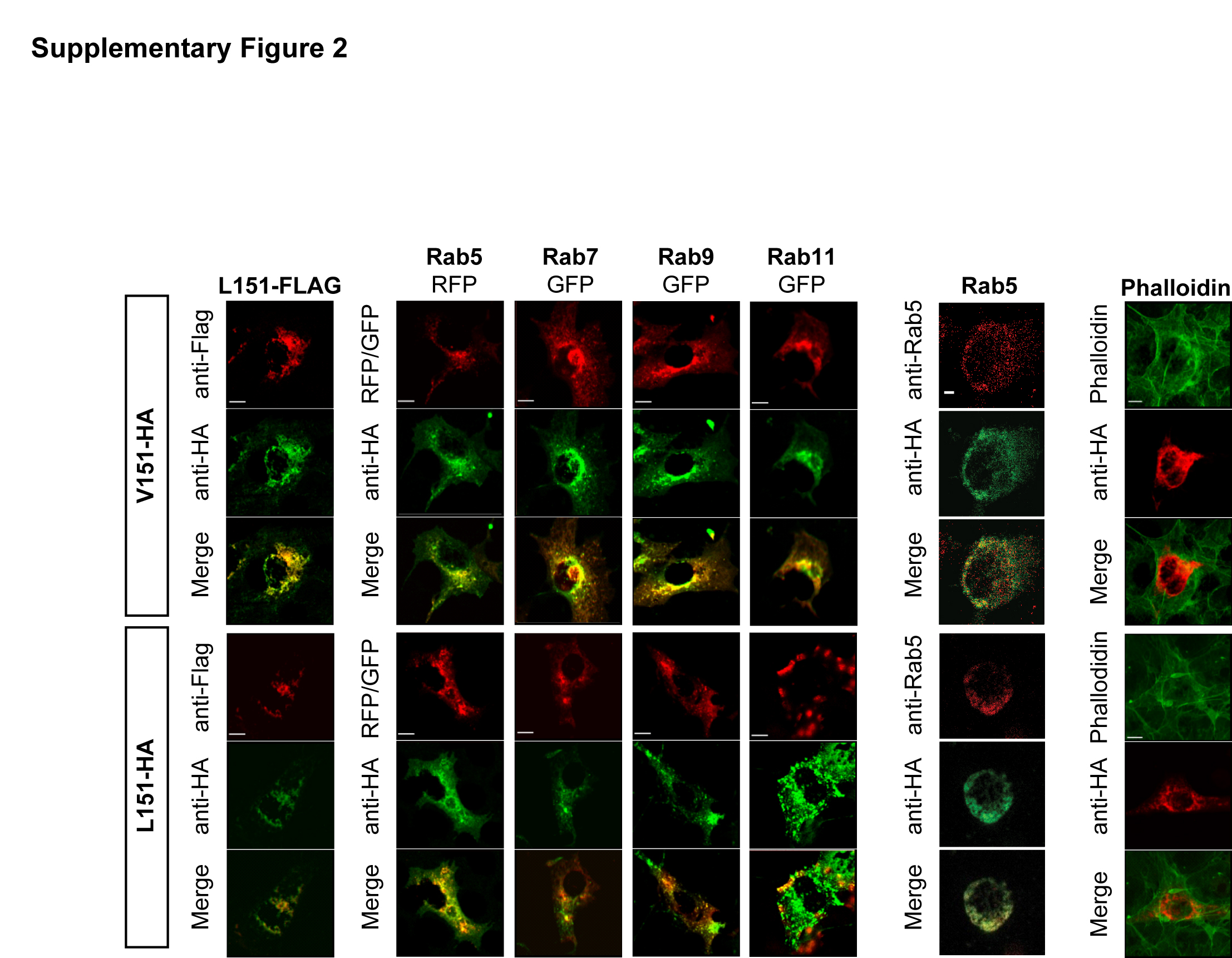


**Supplementary Fig. S2 Subcellular localization of ADCY2 variants in comparison to different markers of subcellular compartments.** Representative photomicrographs COS-7 cells transiently transfected with HA-tagged variants of ADCY2-V151 (top rows) or ADCY2-L151 (bottom rows). Cells were co-transfected with a FLAG-tagged variant of ADCY2-L151, fluorescently tagged (from left to right) Rab5 (early endosome), Rab7 (late endosome, transport from early to late endosomes and from late endosome lysosome), Rab9 (late endosome, transport from late endosomes to the trans-Golgi network) and Rab11 (recycling endosome) or stained against endogenous Rab5 and phalloidin (cell membrane). The detailed quantification of the co-localization of ADCY2 variants with each other and with makers for different subcellular compartments are depicted in Figure 2g-j.


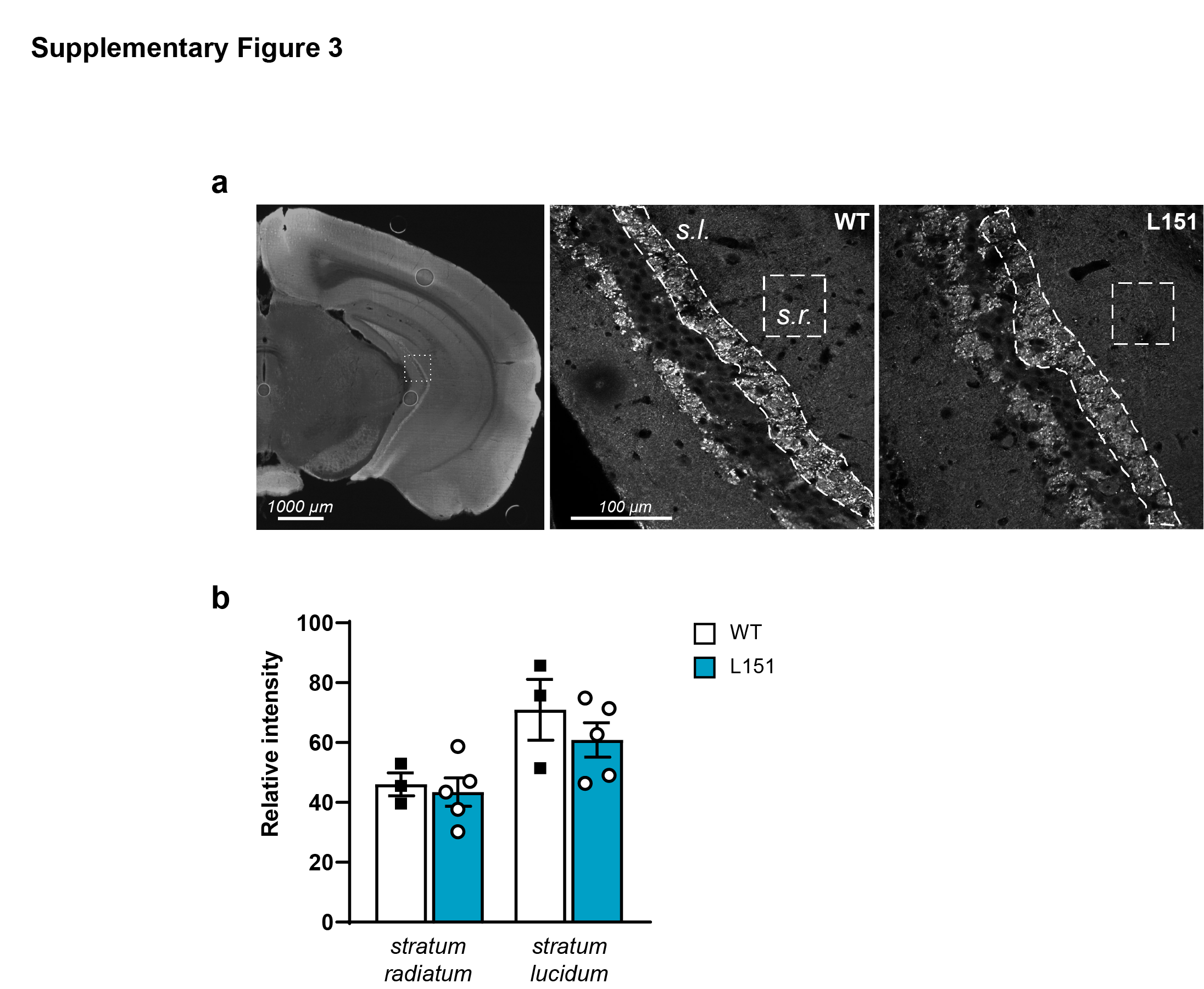


**Supplementary Fig. S3 Quantification of synapsin 1 (SYN1) expression in the ventral hippocampus. a** Right, overview showing the ventral hippocampus and the area used for quantification. Left, higher magnifications depicting the regions in the *stratum radiatum* (*s.r.*) and *stratum lucidum* (*s.l.*) which were used for quantification. **b** Quantification of SYN1 expression.


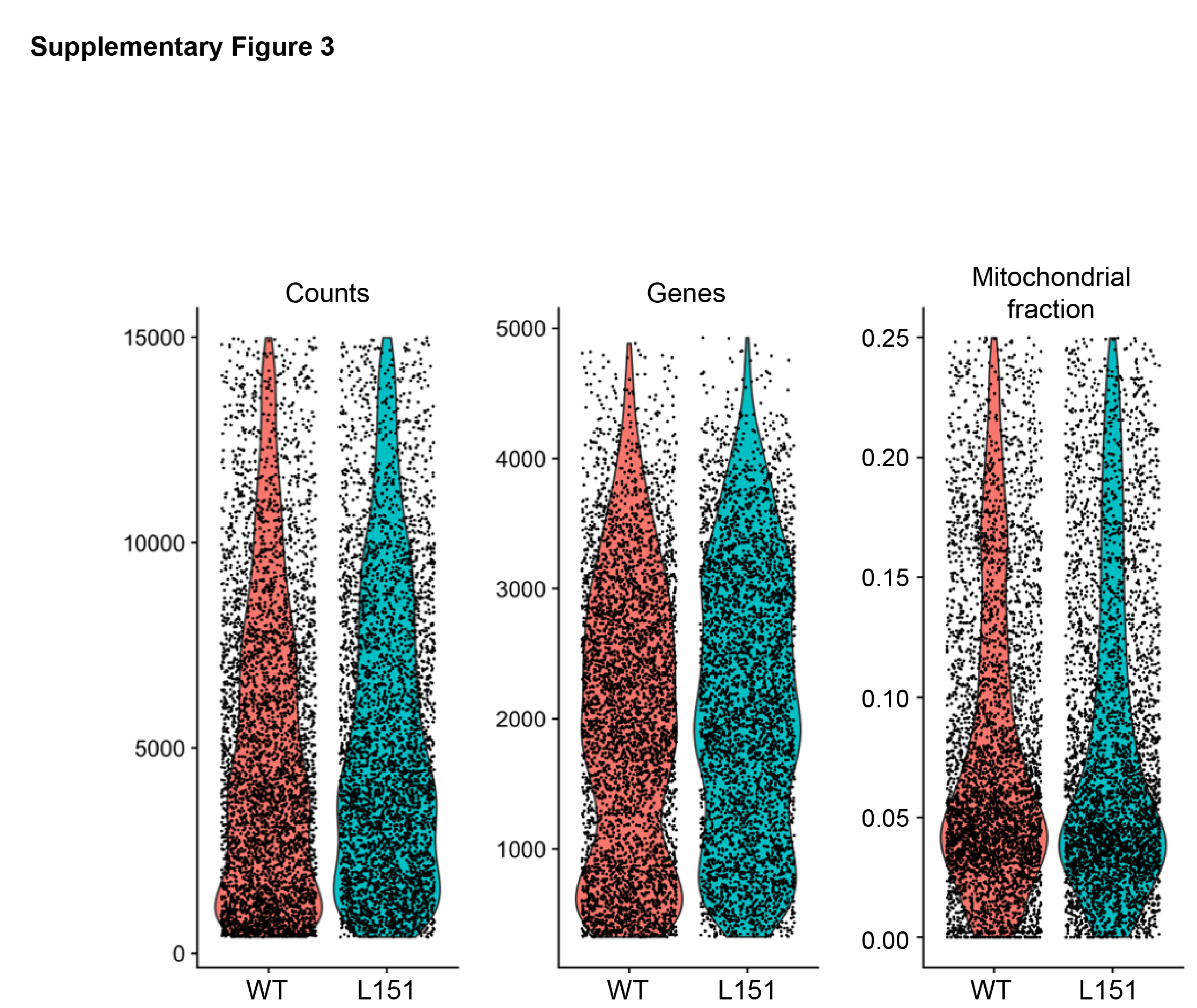


**Supplementary Fig. S4 Quality Control (QC) plots.** Violin plots showing the distribution of the number of counts, genes, and the percentage of mitochondrial fraction in the vHPC subjected to scRNA-Seq.


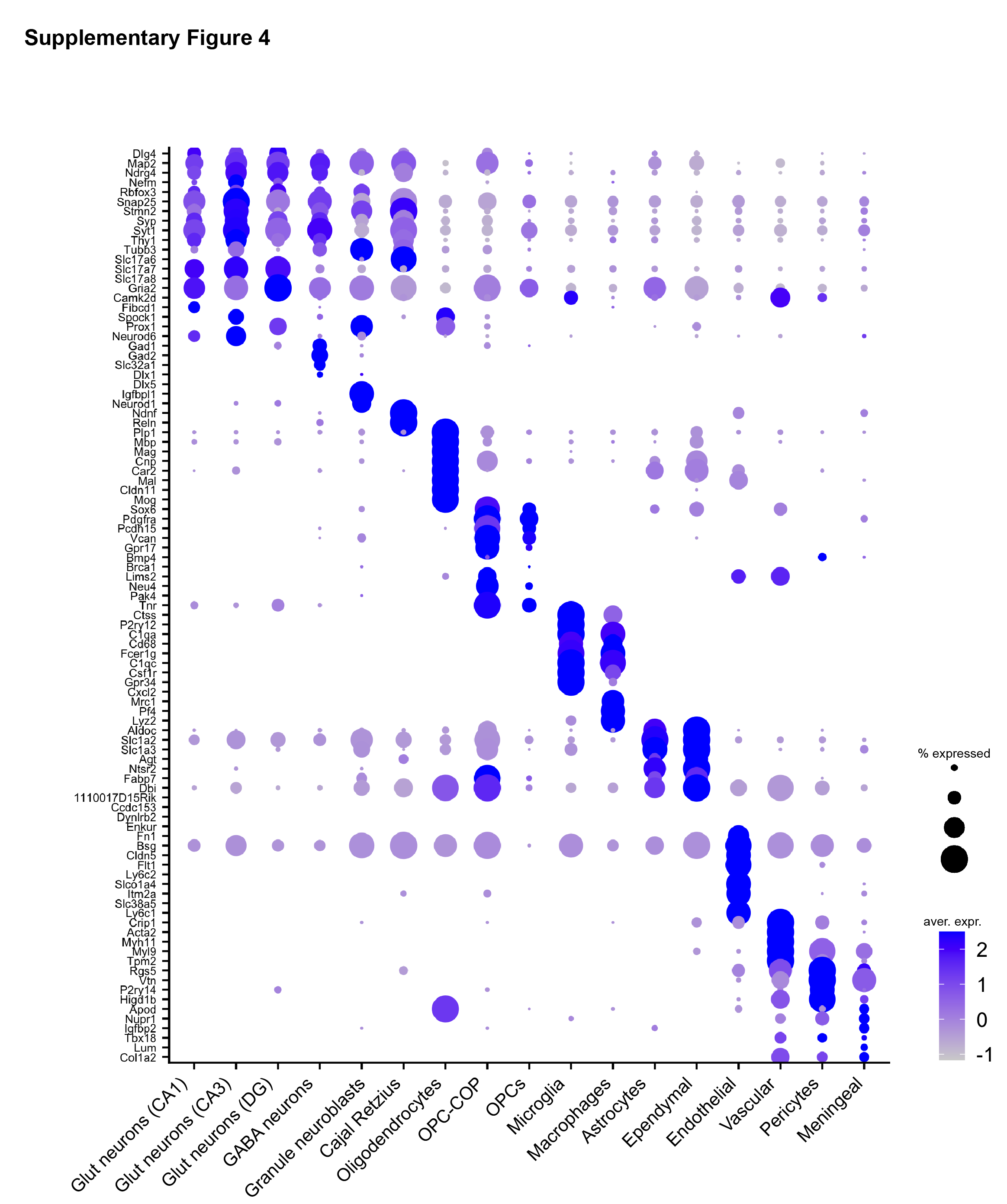


**Supplementary Fig. S5 Identification of cell clusters in the ventral hippocampus.** The plot shows marker genes (left) for each cell cluster (bottom), with the size of the dot corresponding to the percentage of cells within the cell population expressing the respective gene. The brightness of the color represents the average expression level across all cells within the cluster (dark blue: expression = 2, light grey: expression = -1).

**
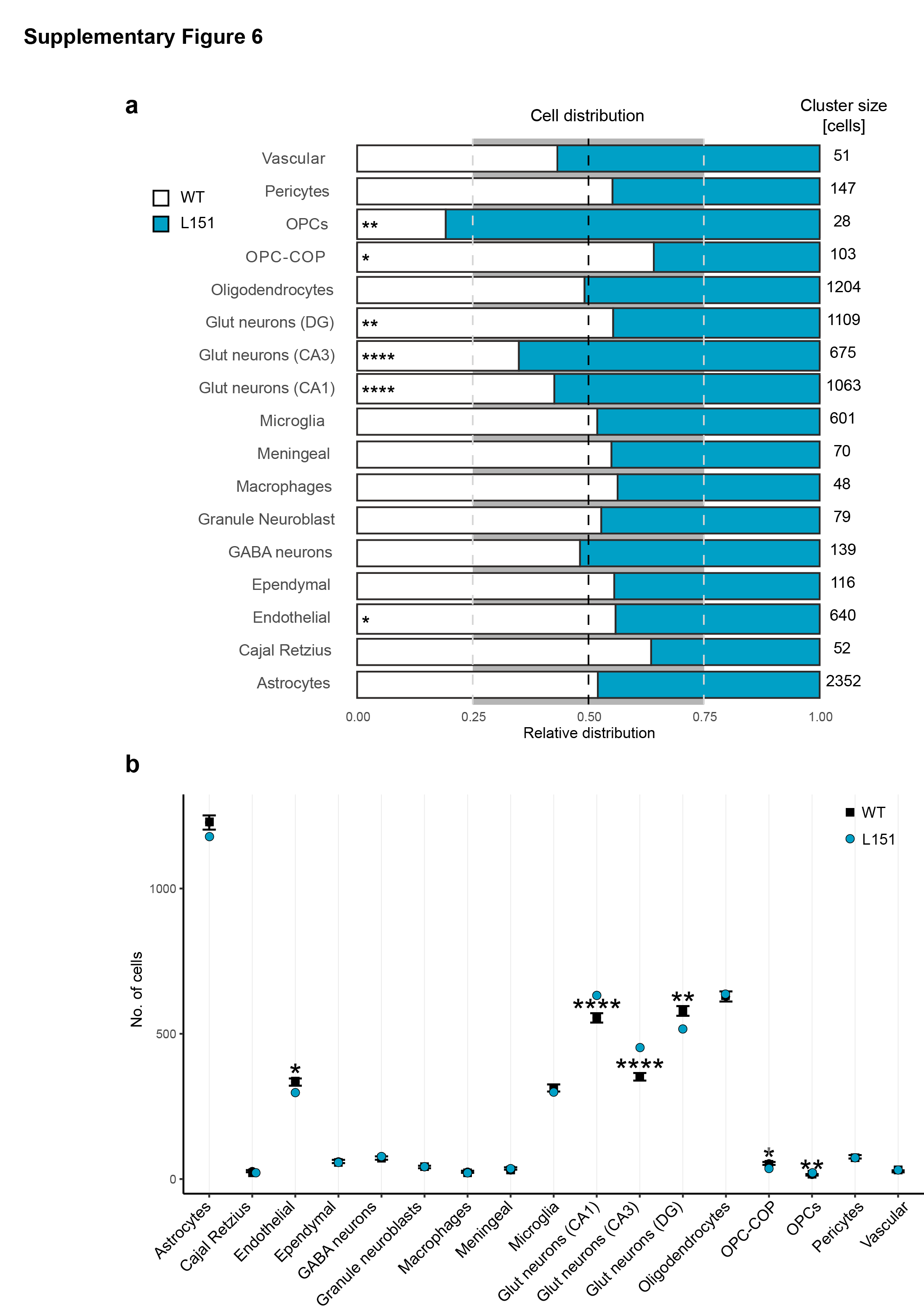
**

**Supplementary Fig. S6 Analysis of the distribution of WT- and L151-derived cells in each cluster. a** Bar plot showing normalized distributions of cells from WT and L151 mice in each cluster analyzed by Fisher t-test. The number of cells in each cluster is provided at the right. **b** Graphical visualization of the absolute numbers of WT and L151 cells in each cluster analyzed by binominal test. Both tests revealed that clusters of endothelial cells, glut neurons (CA1, CA3, DG), OPC-COP and OPCs deviate from the expected balanced distribution. * p < 0.05, ** p < 0.01, p < 0,0001.

**Supplementary Table 1**

Predicted genomic off-targets of sgAdcy2-a and sgAdcy2-b

|  | **Target sequence PAM** | **Chromosome** | **Position*** | **Direction** | **Localization** | **Algorithm** |
| --- | --- | --- | --- | --- | --- | --- |
| **sgAdcy2-a** |  |  |  |  |  |  |
| On target | CCATGCTTCCCTTCAACATG AGG | Chr13 | 69,036,182 | - | *Adcy2* | *CRISPRscan & Cas-OFFinder* |
| Off-a1 | CCATGCTTgCCTTtAACATG AGG | Chr2 | 126,265,547 | + | *Atp8b4* | *CRISPRscan* |
| Off-a2 | CCATGCTTCCCTTCAACAcc AGG | Chr13 | 31,462,773 | - | *intergenic* | *CRISPRscan & Cas-OFFinder* |
| Off-a3 | CCATcCTTCCCTTCAACATa AGt | Chr17 | 27,770,978 | + | *intergenic* | *CRISPRscan* |
| Off-a4 | CtcTGCTTCCCTTCAACATG CtG | Chr17 | 43,047,680 | - | *Adgrf2* | *CRISPRscan* |
| Off-a5 | CCATGCTTCCCTTCcACAgG CtG | Chr17 | 45,423,118 | + | *Supt3* | *CRISPRscan* |
|  |  |  |  |  |  |  |
| **sgAdcy2-b** |  |  |  |  |  |  |
| On target | AGGAAGAATGACACCTGTGG AGG | Chr13 | 69,036,225 | + | *Adcy2* | *CRISPRscan & Cas-OFFinder* |
| Off-b1 | gGGAAGAATGACtCCTGTGG TGG | Chr5 | 73,265,885 | + | *Fryl* | *CRISPRscan & Cas-OFFinder* |
| Off-b2 | AGGAAGAATGAgACCTGgGG CaG | Chr5 | 144,090,815 | + | *Lmtk2* | *CRISPRscan* |
| Off-b3 | AGGAAGAATGACACCTGaGG TtG | Chr6 | 91,429,743 | + | *intergenic* | *CRISPRscan* |
| Off-b4 | AGGAAGAATGcCACCTGaGG TGt | Chr8 | 11,9438,925 | + | *Cdh13* | *CRISPRscan* |
| Off-b5 | AGGAAGAAaGACACCTGaGG CaG | Chr11 | 89,001,172 | - | *intergenic* | *CRISPRscan* |
| Off-b6 | AGGAAtgATGACACCTGTGG GtG | Chr13 | 40,367,415 | - | *Ofcc1* | *CRISPRscan* |
| Off-b7 | AGGAAGAAccACACCTGTGG AGa | Chr13 | 54,481,869 | + | *Gm48622* | *CRISPRscan* |
| Off-b8 | AGGAAGgATGACACCTGaGG GcG | Chr14 | 22,619,458 | + | *Lrmda* | *CRISPRscan* |
| Off-b9 | AGGAAGAATGACACCTGaGG TtG | Chr19 | 47,605,666 | + | *Slk* | *CRISPRscan* |
| Off-b10 | AGGAgGAATGACACCTGaGG TGG | ChrX | 70,606,891 | + | *intergenic* | *CRISPRscan &Cas-OFFinder* |

Used algorithms: CRISPRscan (<https://www.crisprscan.org/>), Cas-OFFinder (<http://www.rgenome.net/cas-offinder>). * Positions according to Ensembl genome assembly GRCm39. The PAM sequences (NGG) is shown in blue color and mismatched sequence to each on-target site are shown in red color respectively.

**Supplementary Table S2**

**Genes differentially expressed in cell types of the vHPC of WT and L151 mice.**

| **Cell type** | **Gene** | **Normalized V151 expression** | **Normalized L151 expression** | **Log FC average** | **Padj.** | **Significance** |
| --- | --- | --- | --- | --- | --- | --- |
| Glut (CA1) | Gm42418 | 5.31461 | 6.05473 | 0.740121 | 1.44E-25 | TRUE |
| Glut (CA1) | Hsp90aa1 | 2.27363 | 2.73308 | 0.459449 | 0.019431 | TRUE |
| Glut (CA1) | Snca | 1.82011 | 2.24271 | 0.422601 | 0.016951 | TRUE |
| Glut (CA1) | Enc1 | 1.22320 | 1.64196 | 0.418761 | 0.000254 | TRUE |
| Glut (CA1) | Hsp90ab1 | 2.84851 | 3.24240 | 0.39389 | 0.001983 | TRUE |
| Glut (CA1) | Calm2 | 3.17556 | 3.54490 | 0.369343 | 0.004374 | TRUE |
| Glut (CA1) | Calm1 | 3.20626 | 3.53475 | 0.328484 | 0.001032 | TRUE |
| Glut (CA1) | Atp2a2 | 1.77298 | 2.01763 | 0.244653 | 4.02E-05 | TRUE |
| Glut (CA1) | App | 2.13670 | 2.36974 | 0.23304 | 9.82E-06 | TRUE |
| Glut (CA1) | Serinc1 | 2.09103 | 2.30634 | 0.215311 | 0.006126 | TRUE |
| Glut (CA1) | Gpm6a | 2.42894 | 2.58795 | 0.159013 | 0.001541 | TRUE |
| Glut (CA1) | Atp1b1 | 2.95872 | 3.10962 | 0.150901 | 2.59E-05 | TRUE |
| Glut (CA1) | Rtn1 | 3.21689 | 3.34911 | 0.132219 | 0.032091 | TRUE |
| Glut (CA1) | Mbtd1 | 0.64469 | 0.37050 | -0.27419 | 0.006071 | TRUE |
| Glut (CA1) | Prkca | 1.18482 | 0.85967 | -0.32515 | 0.041875 | TRUE |
| Glut (CA1) | Epha7 | 1.15303 | 0.82547 | -0.32756 | 0.009097 | TRUE |
| Glut (CA1) | Gm15594 | 0.73569 | 0.37114 | -0.36455 | 0.00034 | TRUE |
| Glut (CA1) | Ubn2 | 1.15717 | 0.78599 | -0.37118 | 0.031318 | TRUE |
| Glut (CA1) | Gm12027 | 0.66474 | 0.27943 | -0.38531 | 0.00015 | TRUE |
| Glut (CA1) | Gm26699 | 0.94061 | 0.53676 | -0.40385 | 0.005019 | TRUE |
| Glut (CA1) | Meg3 | 5.04816 | 4.57482 | -0.47334 | 0.010889 | TRUE |
| Glut (CA1) | Trank1 | 1.30848 | 0.82321 | -0.48527 | 0.000172 | TRUE |
| Glut (CA1) | Malat1 | 6.99968 | 6.43310 | -0.56658 | 0.001658 | TRUE |
| Glut (CA1) | 4921539H07Rik | 1.13232 | 0.44820 | -0.68412 | 1.83E-05 | TRUE |
| Glut (CA1) | AC149090.1 | 2.17399 | 1.35755 | -0.81644 | 4.11E-18 | TRUE |
| Oligodendrocytes | Gm42418 | 4.50359 | 4.71878 | 0.215192 | 2.07E-13 | TRUE |
| Oligodendrocytes | Sec11c | 2.58382 | 2.70275 | 0.118933 | 0.029826 | TRUE |
| Oligodendrocytes | Chchd2 | 2.36859 | 2.25178 | -0.1168 | 0.002963 | TRUE |
| Oligodendrocytes | Jam3 | 1.66280 | 1.54110 | -0.1217 | 0.043961 | TRUE |
| Oligodendrocytes | Kctd13 | 2.35166 | 2.19675 | -0.15491 | 0.000102 | TRUE |
| Oligodendrocytes | Fkbp4 | 1.06409 | 0.90590 | -0.15819 | 0.019688 | TRUE |
| Oligodendrocytes | Dnajb6 | 1.02632 | 0.86580 | -0.16052 | 0.03382 | TRUE |
| Oligodendrocytes | Ahsa1 | 0.95550 | 0.79261 | -0.16289 | 0.001928 | TRUE |
| Oligodendrocytes | Sez6l2 | 2.10945 | 1.92597 | -0.18348 | 9.78E-05 | TRUE |
| Oligodendrocytes | Pdia6 | 1.04105 | 0.85728 | -0.18377 | 0.032635 | TRUE |
| Oligodendrocytes | Mt2 | 0.36342 | 0.17810 | -0.18532 | 0.02028 | TRUE |
| Oligodendrocytes | Hsp90aa1 | 3.33883 | 3.13849 | -0.20034 | 7.47E-11 | TRUE |
| Oligodendrocytes | Hspa5 | 1.75885 | 1.55603 | -0.20282 | 0.007808 | TRUE |
| Oligodendrocytes | Hsp90ab1 | 3.51641 | 3.31312 | -0.2033 | 2.18E-18 | TRUE |
| Oligodendrocytes | Hspa4l | 0.72601 | 0.51565 | -0.21036 | 0.00019 | TRUE |
| Oligodendrocytes | Pdia3 | 1.43803 | 1.22741 | -0.21062 | 1.03E-06 | TRUE |
| Oligodendrocytes | Ndrg2 | 0.96365 | 0.74377 | -0.21989 | 0.000912 | TRUE |
| Oligodendrocytes | Tmsb10 | 0.52028 | 0.28892 | -0.23136 | 0.023249 | TRUE |
| Oligodendrocytes | Sgk1 | 1.93676 | 1.64915 | -0.28761 | 6.46E-06 | TRUE |
| Oligodendrocytes | Calr | 1.66547 | 1.32866 | -0.33682 | 3.99E-20 | TRUE |
| Astrocytes | Gm42418 | 5.45490 | 5.77914 | 0.32424 | 3.53E-27 | TRUE |
| Astrocytes | Eps8 | 0.73291 | 0.94879 | 0.215881 | 0.018709 | TRUE |
| Astrocytes | Ifitm2 | 0.22702 | 0.38696 | 0.159939 | 0.017109 | TRUE |
| Astrocytes | Hspa8 | 2.40428 | 2.23349 | -0.17079 | 0.001091 | TRUE |
| Astrocytes | Stmn3 | 1.11702 | 0.92905 | -0.18796 | 0.009171 | TRUE |
| Astrocytes | Ndufc1 | 1.21121 | 0.99146 | -0.21976 | 0.025325 | TRUE |
| Astrocytes | Insig1 | 0.97655 | 0.73071 | -0.24584 | 4.00E-05 | TRUE |
| Astrocytes | Pcyt2 | 1.03382 | 0.77106 | -0.26276 | 1.26E-05 | TRUE |
| Astrocytes | Hspa5 | 1.50081 | 1.23352 | -0.26729 | 4.26E-09 | TRUE |
| Astrocytes | Mt1 | 3.85916 | 3.59142 | -0.26774 | 3.64E-06 | TRUE |
| Astrocytes | AC149090.1 | 0.89808 | 0.59601 | -0.30206 | 6.08E-07 | TRUE |
| Glut (DG) | Camk2b | 2.10619 | 2.41656 | 0.310369 | 1.01E-10 | TRUE |
| Glut (DG) | Slc17a7 | 2.16122 | 2.38188 | 0.22066 | 9.00E-06 | TRUE |
| Glut (DG) | Fth1 | 3.21446 | 3.43111 | 0.216643 | 0.007164 | TRUE |
| Glut (DG) | Gm42418 | 4.95298 | 5.14414 | 0.191166 | 1.16E-16 | TRUE |
| Glut (DG) | Slc15a2 | 0.38987 | 0.09016 | -0.29972 | 7.86E-08 | TRUE |
| Glut (DG) | AC149090.1 | 2.24483 | 1.85160 | -0.39323 | 3.67E-19 | TRUE |
| Endothelial | Lmo2 | 1.64169 | 1.91948 | 0.277796 | 0.005055 | TRUE |
| Endothelial | Nr1d1 | 0.2230 | 0.47364 | 0.250598 | 0.040201 | TRUE |
| Endothelial | Hspb1 | 3.14208 | 2.84901 | -0.29306 | 0.001935 | TRUE |
| Endothelial | Mt1 | 2.15184 | 1.60771 | -0.54413 | 4.02E-07 | TRUE |
| Glut (CA3) | Gm42418 | 4.94123 | 5.39923 | 0.457998 | 2.97E-13 | TRUE |
| Glut (CA3) | Fxyd7 | 1.42719 | 0.91807 | -0.50912 | 0.00784 | TRUE |
| Microglia | Gm42418 | 4.90566 | 5.23869 | 0.333031 | 3.67E-24 | TRUE |
| OPC-COP | Calr | 2.33508 | 1.78638 | -0.54871 | 0.024944 | TRUE |

FC: fold change; Padj.: adjusted P value
